# Optimizing phosphorus released in calcareous soil amended with bone char and bone ash using response surface methodology and desirability function

**DOI:** 10.1038/s41598-025-13548-5

**Published:** 2025-08-18

**Authors:** Yasser A. El-Damarawy, Eman M. Saleh, Omar M. Ibrahim, Ahmed A. El-Refaey, Maher E. Saleh, Eman H. El-Gamal

**Affiliations:** 1 Soils and Water Use Department, National Research Centre, Cairo, Egypt; 2 Department of Soils and Water Sciences, Faculty of Agriculture, Alexandria University, El-Shatby, 21545 Alexandria Egypt; 3 Plant Production Department, Arid Lands Cultivation Research Institute (ALCRI), City of Scientific Research and Technological Applications (SRTA-City), New Borg El-Arab City, 21934 Alexandria Egypt; 4 Soil and Water Science Department, Faculty of Desert and Environmental Agriculture, Matrouh University, Fuka, Egypt; 5 Land and Water Technologies Department, Arid Lands Cultivation Research Institute (ALCRI), City of Scientific Research and Technological Applications (SRTA- City), New Borg El Arab City, 21934 Alexandria Egypt

**Keywords:** Bone ash, Bone char, Central composite design, Phosphorus levels, Incubation time, Calcareous soils, Soil fertility, Environmental sciences, Chemistry, Materials science

## Abstract

This study evaluated the optimization of phosphorus (P) released in calcareous soil-amended with bone ash (BA) and bone char (BC) compared to phosphate rock (PR) and single superphosphate (SSP) using a central composite design and desirability function. Incubation experiment was conducted (≃30 °C) to investigate the effects of P-levels (PL, 1000–4000 mg P/kg soil) and incubation time (14–90 days) on available-P, soluble-P, -Ca²⁺, -Mg²⁺, and soil-pH. Wetting and drying cycles were applied using distilled water (DW) and acidified water (AW). Results revealed a significant increase in soil phosphorus availability and solubility over time, particularly at higher PL. AW was more effective than DW in increasing available-P and soluble-P, -Ca²⁺, and -Mg²⁺, while reducing soil-pH and incubation time. Regardless of SSP behavior due to its high solubility, maximum amounts of available-P in BA-, BC-, and PR-amended soil for DW application were 40.92, 31.53, and 16.74 mg/kg at 40.6, 55.8, and 14 days, respectively, with PL of 3850 mg/kg for BA and 4000 mg/kg for BC and PR. For soluble-P, maximum amounts were 3.1, 2.62, and 0.85 mg/kg with AW application after incubation times of 36.8, 63.4, and 90 days, respectively, which were higher than DW application. BA-amended soil with DW and AW applications exhibited optimal responses for available and soluble phosphorus, higher than BC-amendment. PL and incubation time interactions were pronounced in enhancing the studied parameters. The study concluded that AW, simulating rhizosphere acidification, significantly optimized P-release and related parameters compared to DW. This perspective requires further research for broader applications, especially for its applications in large-scale agricultural systems. Generally, BA and BC redistributed the phosphorus solubility and availability in soil, subsequently enhancing soil fertility and sustainability as an eco-friendly phosphate fertilizer.

**Supplementary Information:**

The online version contains supplementary material available at 10.1038/s41598-025-13548-5.

## Introduction

Globally, calcareous soils cover approximately 30% of the Earth’s outermost layer and are characterized by the presence of free carbonates in the form of calcium carbonate (CaCO₃) or calcium magnesium carbonate (CaMg(CO₃)₂), with concentrations exceeding 10%^1,2^. These soils mainly originate in arid and semi-arid regions, where limited rainfall promotes the accumulation of carbonate salts within the soil profile, forming caliche layers^1,3^. In Egypt, calcareous soils occupy about 25–30% of the total land area. These soils are commonly found in regions bordering the Nile Valley’s fringe zone, where CaCO₃ ranges from moderate (3%) to high (30%). Beyond this zone, extending to eastern and western deserts, the carbonate content increases significantly, reaching between 30% and 80%^1,4^. The physicochemical properties of calcareous soils are significantly affected by high levels of carbonates, which affect soil-water retention, surface crusting, and nutrient availability^5^. Notably, approximately 80–90% of soil phosphorus becomes unavailable to plants in these soils (with a pH above 8). This is due to the precipitation of phosphorus as insoluble calcium phosphate (Ca-P) compounds, such as hydroxyapatite^6,7^.

Phosphorus (P) is a critical macronutrient necessary for plant growth and food production, significantly playing a vital role in numerous physiological and biochemical functions, including DNA synthesis, energy generation (ATP and ADP), photosynthesis, and respiration^8–11^. Nevertheless, insufficient phosphorus supply limits plant growth by reducing photosynthesis activity and total biomass accumulation, which negatively affects the overall life cycle of plants^12,13^. It is estimated that P deficiency reduces the crop production on 30–40% of the world’s arable land^14^. Globally, the agriculture industry is the largest sector, consuming approximately 90% of the phosphorus requirements^15^. It is well recognized that the primary raw material used for phosphate fertilizer production is phosphate rock, a non-renewable material expected to be depleted within the following 50 or 100 years^16^. Therefore, phosphate rock must be replaced in the phosphate fertilizer industry with an alternative and sustainable solution. This approach would guarantee the continuous production of phosphorus fertilizers for agricultural ecosystems and environmental preservation^16,17^.

Application of soluble phosphorus fertilizer in the form of phosphorus pentoxide (P_2_O_5_) is a quick and conventional method that transforms phosphorus into insoluble forms in alkaline soils. Notably, this practice leads to the extreme and repeated application of phosphorus fertilizers to cultivated soil^18,19^. These additions cause various environmental issues, such as the eutrophication phenomenon, due to the phosphorus loss and leakage through drainage or surface runoff into marine and aquatic environments. Additionally, phosphate fertilizers may contain trace amounts of toxic heavy metals (e.g., Cd, Ni, Cr, As, Pb, Se, Cu). These contaminants can negatively affect the behavior of soil microbial activity, plant biological processes, and some nutrient availability. Moreover, these toxic metals pose harmful effects on humans, which leads environments to break down in the long term^20^. Annually, P-fertilizer application rates are approximately 120 kg P/ha^1^, depending on the crop species, production levels, and management systems^21,22^.

It is recognized that various organic wastes, including sewage sludge, animal manure, bones, blood, and crop residues, contain large amounts of phosphorus. Consequently, alternative P-Sources in agricultural soils are necessary to avoid contamination risks associated with heavy metals^8,23,24^. As identified, appetite minerals are widely distributed in igneous rocks as natural sources of phosphorus. It was found that the main component in animal bones is biological apatite (Bioapatite) and that most of its composition is organic phosphorus, which is an almost crystalline structure of calcium phosphate. Interestingly, the animal bone ash composite contains calcium (25–29% Ca) and phosphorus (15–19% P) elements closely in the same range that were found in the phosphate rock (35% Ca and 15% P)^25^. The thermal decomposition process of animal bone in an inert condition of air “pyrolysis process” produces bone char consisting mainly of hydroxyapatite (HAP) (70–76%), calcium carbonate (7–9%), and amorphous carbon (9–11%)^17,26^. Remarkably, the most important benefit of both bone ash and bone char is that they have an insignificant concentration of toxic metals relative to phosphate rock^8,22^.

One of the most effective optimization methods is Response Surface Methodology (RSM), a statistical method of modeling both linear and quadratic effects, enabling the optimization of resources and responses^27^. In RSM, a response can be maximized, minimized, or achieved a target value at the desired optimum levels of the input variables. Additionally, RSM is capable to estimate the interaction effects of several factors simultaneously with a limited number of experimental runs. Thus, RSM is a powerful tool in statistical modeling and optimization of the inputs with a minimum number of experimental runs. Central composite design (CCD) is one of the designs of RSM which is considered an alternative to the full factorial design (FFD). The CCD design was developed by Box and Wilson^28^ and enhanced by Box and Hunter^29^, it gives almost the same information as a three-level full factorial design by a lower number of experimental runs. Desirability function (DF) is a popular strategy for multiple response optimization. The DF scale ranges from 0 to 1, indicating how near the response is to its optimum value. Numerical optimization identifies a region where the DF was maximized, minimized, or targeted^30^.

Phosphorus solubility and availability of applied fertilizers in agricultural soils, particularly calcareous soil, are often controlled depending on soil physicochemical properties as well as the nature of applied fertilizers. The objectives of this study were to focus on optimizing soluble and available phosphorus in calcareous soils amended with nontraditional P-Sources like bone char (BC) and bone ash (BA) in comparison with the traditional P fertilizers. We hypothesize that despite high P availability due to the addition of phosphate fertilizers, several interactions with soil components over time fix this nutrient in the soil, which makes it unavailable during the growing season. Therefore, the addition of nontraditional P-Sources as slow-release fertilizers to meet the phosphorus requirements of growing plants on time. The specific objectives of this study are as follows:

1. Evaluate P solubility and availability over time in calcareous soil amended with varying rates of bone ash and bone char compared to PR and SSP using distilled or acidified water during wetting-drying cycles.
2. Assess the factors affecting P release in calcareous soil amended with different P-Sources under investigated conditions, such as soluble Ca²⁺ and Mg²⁺ concentrations and changes in soil pH.

## Materials and methods

### Physicochemical characterization of phosphorus sources and soil collection

Cattle bone waste materials were collected from local slaughterhouses, then cleaned from meat and fat residues and rinsed several times with distilled water. The cleaned bone materials were air-dried and divided into two parts to convert into bone char and bone ash. The bone char (BC) was produced through a slow pyrolysis process using a primitive pyrolytic unit at a temperature around 500 °C for 3 hr^31^. Bone ash (BA) was obtained by thermo-combusting the bone in a muffle furnace at 800 °C for 2 h in an open porcelain crucible. Commercial phosphate rock (PR) was purchased from El-Nasr Mining Company, Aswan, Egypt. Additionally, single superphosphate (SSP) fertilizer was obtained from the Agricultural Research Station, Faculty of Agriculture, Alexandria University, as a common phosphorus fertilizer source used in Egypt. All the mentioned P-Sources were crushed, sieved through a 0.5 mm sieve, and stored in polyethylene bottles for further use.

Total concentrations of minerals (Ca, Mg, Cd, Cu, Mn, Cr, Pb, Ni, and Zn) were determined in a clear solution of digested samples with HNO_3_ and H_2_O_2_ at 350 ℃ for 2 h using Atomic Absorption Spectrophotometer, Varian, Spectra AA-220^32^. Total phosphorus was determined in the digested solution by ammonium paramolybdate-vanadate method, and the yellow color intensity was measured at 420-nm wavelength by T80 UV/VIS Spectrophotometer, PG Instruments Ltd^33^. CHNS analyzer was used to determine the total carbon, hydrogen, nitrogen, and sulfur content by (Vario MACRO cube, Elementar, Germany). The X-ray diffraction (XRD) was performed using a SHIMADZU XRD-7000, X-ray diffractometer with Cu-Kα radiation operated at 30 kV and 30 mA and scanning range from 4 to 70° 2θ, using a step size of 0.02° 2θ and a scan speed of 4° min^- 1^. The surface morphology of the four phosphorus source materials was examined by scanning electron microscopy (SEM) at a magnification of X1000 using a Joel 6360 OLA. In order to avoid the build-up of local electrical charges, the samples were coated with gold using a sputtering coater (S150B, Edwards High Vacuum Ltd., UK) before investigation. Additionally, surface functional groups of obtained materials were identified by Fourier-Transform Infrared (FTIR) spectroscopy, recorded in the wavelength range of 4000–400 cm^-1^ using JASCO FT/IR-5300 (JASCO Corporation, Japan). The surface area of the mentioned P-Sources materials was measured using N_2_ sorption isotherms at 77 °K. run on BElSORP-mini II instrument and the data were analyzed according to Brunauer-Emmett-Teller (BET) method.

The experimental soil was calcareous (0–30 cm, 32.4% CaCO_3_) collected from the Experimental Farm of the City of Scientific Research and Technological Applications (SRTA-City) located at Old Borg El-Arab City, Alexandria, Egypt (30° 53 × 33.17” N, 29° 22 × 46.43” E). Soil characterization was illustrated in Table (1), following Page et al. methods^34^. The soil pH was alkaline (40% soil-water suspension, 8.35), and its salinity of soil paste extraction was categorized as moderately saline (EC, 2.27 dS/m)^35^. Additionally, it had a high content of total carbonate measured by the calcimeter method with a moderate level of organic matter (1.09%) determined by the wet oxidation method. Available nitrogen (128 mg/kg) extracted by neutral KCl (1.0 N) and phosphorus (Olsen method, 5.00 mg/kg) were in a low range. In contrast, available potassium measured in NH_4_OAc (1 N, pH 7) was found to be high^36,37^. The total phosphorus (TP) content was 0.43 g/kg, meaning that the plant-available phosphorus was 1.16%, based on the measured amount of available phosphorus; the TP was determined in the clear acid extraction of the Na_2_CO_3_ fusion method, and the formed color density was measured as illustrated above. The particle size distribution was 18.70% clay, 16.00% silt, and 65.30% sand, with a sandy-loam texture and a water-holding capacity (WHC) equal to 50%. According to the Soil Survey Staff, the study area primarily features soils belonging to the Aridisols order and classified as Typic Haplocalcids^38^.

**Table 1:** Some soil physicochemical properties.

Sand	Silt	Clay	pH	EC	O.M	CaCO_3_	Available Macronutrients			Total P
							N	P	K	
%	%	%	---	dS/m	%	%	mg/kg	mg/kg	mg/kg	g/kg
65.3	16.0	18.7	8.35	2.27	1.09	32.40	128	5.00	264	0.43

EC: electric conductivity; O.M: Organic matter.

### Incubation experiment and analyses

A wetting and drying incubation experiment was carried out to study the changes in solubility and availability of phosphorus in the calcareous soil treated with BC, BA, PR, and SSP. A central composite design (CCD) experiment in a completely randomized design (CRD), with three replicates, was conducted. Three levels of applied phosphorus rates (1000, 2500, and 4000 mg P/kg soil) were tested across the four P-Sources. The treated soils were incubated at the laboratory conditions a nearly close temperature of 30 ± 3 ℃ for three time periods (14, 52, and 90 days). The study monitored the effect of these treatments on available P, soluble P, soluble Ca^2+^, soluble Mg^2+^, and soil pH. The experiment was divided into two groups upon wetting type: the first group was wetted with distilled water (DW), while the other was wetted with acidified water (AW) using 0.001 M H_2_SO_4_, (pH 3.02 ± 0.02). Distilled water was used to extract the most labile and mobile P fraction, whereas acidified water simulated rhizosphere acidification. The treated soil was initially wetted with water or acidified water up to 100% of soil water holding capacity (WHC). Then, wetting cycles occurred every six days up to 50% of WHC. After 14-, 52-, and 90-day cycles, soil samples were collected, air-dried, ground, and passed through a 2-mm sieve. The pH was measured in a 1:2.5 (soil: water) suspension after shaking for 2 h. Soluble P, Ca^2+^ and Mg^2+^ were determined in the clear solution of the soil suspension (1:5, w/v). Water-soluble Ca^2+^ and Mg^2+^ were determined by titration method^34^. The available phosphorus was extracted using the Olsen method (0.5 M NaHCO_3_, pH 8.5). Phosphorus amounts in the clear solution, whether soluble or available, was measured depending on blue color density by the ascorbic acid method using spectrophotometer (T80 UV/VIS, PG Instruments Ltd) at a wavelength of 882 nm^39^.

### Central composite design (CCD) layout

In the present study, CCD was used, the face centered type, to study two independent factors (phosphorus levels, PL, 1000–4000 mg P/kg soil; incubation time, 14–90 days). The required number of runs for the CCD consisted of (1) the standard 2^k^ factorial points, where k is the number of factors; (2) 2k points located axially at a distance of α (α = 1 in face centered type) from the center point to estimate the quadratic terms; and (3) replicated runs of the center points to estimate the experimental error. For two factors face centered design, three to five center points are enough^27^. Hence the total number of required experimental runs for the two factors is:

1 $$\:{2}^{\varvec{k}}+2\varvec{k}+{\varvec{C}}_{\varvec{p}}={2}^{2}+2\:\times\:\:2+3=11$$

The CCD was created and analyzed by using package *rsm* R software (version 4.1.0) based on lower and higher levels of incubation time (14 and 90 days) and PL (1000 and 4000 mg P/kg soil); the coded levels were ± 1 for the factorial points and 0 for the center points. The design matrix of the experiment was represented in Table 2 consists of 11 runs expressed as coded and actual levels, where factorial and axial runs were replicated three times. Response surface and contour plots were generated using package *plot 3D* (Plotting multi-dimensional data) version 1.4.1^40^, desirability plots were generated using package *desirability* in R software version 4.3.2^41^.

**Table 2:** Actual and coded levels of the studied factors (incubation time and phosphorus level).

Run No.	Actual levels of factors		Coded levels of factors		Run Type
	Time	PL	x1	x2	
1	14	1000	−1	−1	Factorial
3	14	4000	−1	1	Factorial
6	90	1000	1	−1	Factorial
8	90	4000	1	1	Factorial
2	14	2500	−1	0	Axial
4	52	1000	0	−1	Axial
5	52	4000	0	1	Axial
7	90	2500	1	0	Axial
9	52	2500	0	0	Center
10	52	2500	0	0	Center
11	52	2500	0	0	Center

Time: incubation time/day; PL: phosphorus levels (mg/kg); Factorial and Axial runs are the average of three replicates.

### Mathematical modeling

A full quadratic polynomial model was fitted to predict the measured soil characteristics as functions of incubation time and P level. The full quadratic polynomial equation consists of the following terms: Intercept coefficient ($\:{b}_{0}$), Linear terms ($\:{x}_{1\:}$, $\:{x}_{2}$,…, $\:{x}_{n}$), Quadratic terms ($\:{x}_{1}^{2}$, $\:{x}_{2}^{2}$,…, $\:{x}_{n}^{2}$), Two-way interaction terms ($\:{x}_{1}{x}_{2}$, $\:{x}_{1}{x}_{3}$,…, $\:{x}_{n-i}{x}_{n}$), and Slopes ($\:{b}_{1}$, $\:{b}_{2}$,…, $\:{b}_{n}$).

2 $$\:{\varvec{y}}_{\varvec{i}}=\:{\varvec{b}}_{0}+\:\sum\:_{\varvec{i}=1}^{3}{\varvec{b}}_{\varvec{i}}{\varvec{x}}_{\varvec{i}}+\:\sum\:_{\varvec{i}=1}^{3}{\varvec{b}}_{\varvec{i}\varvec{i}}{\varvec{x}}_{\varvec{i}}^{2}+\:\sum\:_{\varvec{i}=1}^{3}\sum\:_{\varvec{j}=\varvec{i}+1}^{3}{\varvec{b}}_{\varvec{i}\varvec{j}}{\varvec{x}}_{\varvec{i}}{\varvec{x}}_{\varvec{j}}\:$$

where $\:{y}_{i}$ is the predicted response, $\:{b}_{i}$ is the linear term, $\:{b}_{ii}$ is the squared term, $\:{b}_{ij}$ is the interaction term, and $\:{x}_{i}$ and $\:{x}_{j}$ represent the coded factors. In the current experiment, the full quadratic polynomial equation using the uncoded factors was as follows:

3 $$\:{\varvec{y}}_{\varvec{i}}=\:{\varvec{b}}_{0}+\:{\varvec{b}}_{1}{\varvec{x}}_{1}+\:{\varvec{b}}_{2}{\varvec{x}}_{2}+\:{\varvec{b}}_{3}{\varvec{x}}_{3}+\:{\varvec{b}}_{11}{\varvec{x}}_{1}^{2}+\:{\varvec{b}}_{22}{\varvec{x}}_{2}^{2}+\:{\varvec{b}}_{33}{\varvec{x}}_{3}^{2}+\:{\varvec{b}}_{12}{\varvec{x}}_{1}{\varvec{x}}_{2}+\:{\varvec{b}}_{13}{\varvec{x}}_{1}{\varvec{x}}_{3}+\:{\varvec{b}}_{23}{\varvec{x}}_{2}{\varvec{x}}_{3}$$

## Results

### Characteristics of P-Sources

The main physicochemical characteristics of different phosphorus sources (P-Sources) were presented in Table 3. Bone char (BC) exhibited the highest surface area (62.59 m^2^/g) and total pore volume (0.27 cm^3^/g) among all tested P-Sources. While bone ash (BA) had the largest mean pore diameter (72.67 nm), about fourfold compared to the other P-Sources, which may facilitate the nutrient release. It is observed that the total surface area (BET) value of BC increased by 27.21, 2.82, and 3.86 times relative to BA, PR, and SSP, respectively. BA contained the highest phosphorus (P_2_O_5_, 36.08%) and calcium (CaO, 37.84%) proportions, while the greatest content of sulphur was detected in SSP (10%), commonly associated with sulphuric acid during its production^42^. It is reported that SSP contains monocalcium phosphate (MCP), calcium sulphate (CaSO_4_), and phosphorus pentoxide (P_2_O_5_, 16–22%)^43^. On the other hand, BC, BA, and PR had a notable Ca/P ratio (1.68, 1.73 and 1.72, respectively), while SSP showed the lowest ratio (Ca/P, 1.38). Furthermore, bone char was typically enriched in carbon content (C), hydrogen (H), and nitrogen (N), the main element structure of the organic wastes that play a vital role in enhancing soil properties. Additionally, bone char showed a notable concentration of Fe, and Na contents compared to other P-Sources. In contrast, PR had the highest levels of heavy metals, including Cd (25.15 mg/kg), Cu (15.41 mg/kg), Mn (75.66 mg/kg) Pb (27.11 mg/kg) and Zn (171.17 mg/kg).

**Table 3:** Some physical and chemical properties of different P-Sources used in this experiment.

Parameter		Unit	BC	BA	PR	SSP
**Total Surface Area (BET)**		m^2^/g	62.59	2.30	22.20	16.20
**Total Pore Volume p/po**		cm^3^/g	0.27	0.04	0.09	0.07
**Mean Pore Diameter**		nm	17.22	72.67	16.47	17.83
**Total Elements**	P_2_O_5_	%	31.68	36.08	32.28	13.75
	CaO	%	32.2	37.84	33.62	11.52
	Ca/P	Ratio	1.68	1.73	1.72	1.38
	C	%	22.77	0.70	3.03	1.85
	H	%	2.92	0.77	0.72	1.58
	N	%	3.49	0.00	0.00	0.01
	S	%	0.09	0.31	1.73	10.00
	K	%	0.32	1.09	1.18	0.86
	Mg	%	0.36	1.23	0.25	0.48
	Fe	%	20.44	14.86	15.26	13.50
	Na	%	2.59	1.87	1.49	1.74
	Cd	mg/kg	2.22	2.74	25.15	3.36
	Cu	mg/kg	0.12	0.14	15.41	11.01
	Mn	mg/kg	36.13	12.26	75.66	14.03
	Pb	mg/kg	0.08	0.03	27.11	3.14
	Zn	mg/kg	36.28	145.60	171.17	8.43

BC: bone char; BA: bone ash; PR: phosphate rock; SSP: single superphosphate.

**Fig. 1:**
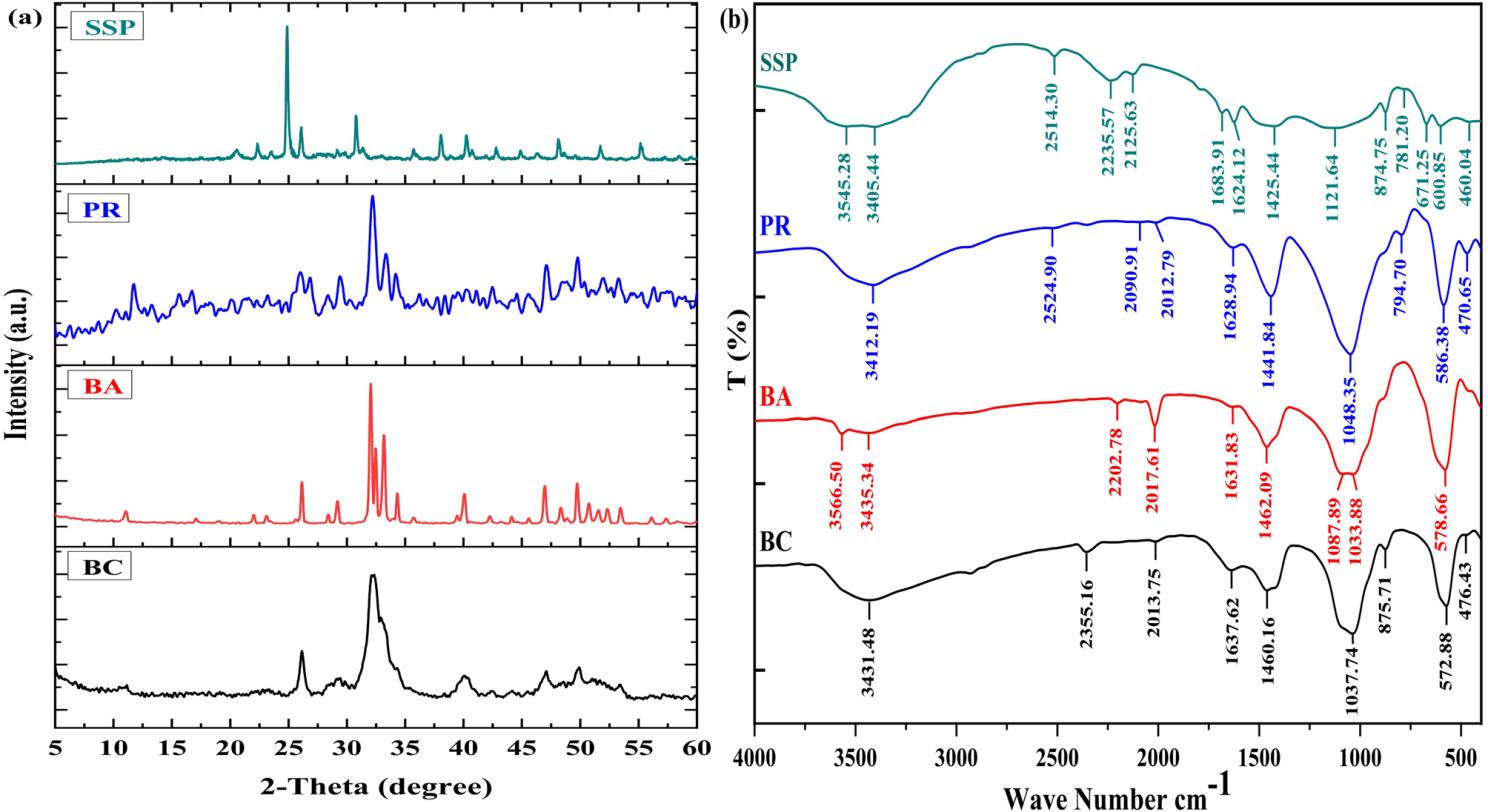
X-ray diffraction (XRD) patterns (**a**) and Fourier transform infrared (FT-IR) spectra (**b**) of the studied phosphorus sources: bone char (BC), bone ash (BA), phosphate rock (PR), and single superphosphate (SSP).

**Fig. 2:**
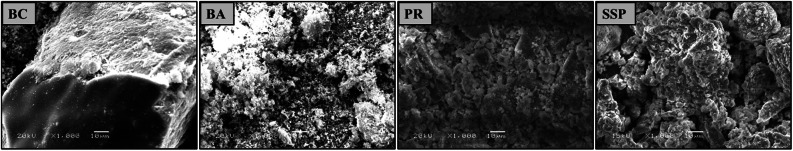
Scanning electron microscopy (SEM) micrographs of studied phosphorus sources at a magnification of X1000: bone char (BC), bone ash (BA), phosphate rock (PR) and single superphosphate (SSP).

The X-ray diffraction (XRD) patterns of bone char (BC), bone ash (BA), phosphate rock (PR), and single superphosphate (SSP) are shown in Fig. 1-a. The diffractograms showed the presence of hydroxyapatite [Ca_10_(PO_4_)_6_(OH)_2_], as confirmed by the diffraction peaks at approximately ≃ 26°, 32°, 40°, 47°, and 49.5° that were presented in all materials. Additionally, peaks corresponding to CaCO_3_ were identified at ≃ 23.0°, 29°, 36°, 48.5°, and 64.7°. The XRD patterns of BA presented similar peaks to BC but showed sharper diffraction peaks compared to those of BC (Fig. 1-a). Furthermore, the diffractograms of SSP exhibited a distinct sharp peak at 27°, attributed to the crystalline phase of gypsum [Ca(SO_4_).2H_2_O]. Figure 1-b showed the Fourier transform infrared (FT-IR) spectra of the different P-Sources obtained from KBr pellets. FT-IR data of BC showed no significant change compared to those of BA and PR concerning the intensity regardless of SSP, which appeared more different. However, the wavelengths of most noted bands in BC have been somewhat changed in association with those recorded for BA. The broad peak ranged from 3435 to 3405 cm^- 1^ was attributed to *v*(O–H) vibration groups^44,45^. Moreover, the vibration broad band of hydroxyl (–OH) stretching was found in BA and SSP at 3566 and 3545 cm^- 1^, respectively. The vibration mode of carbonate groups was absorbed in bands at 1637⁓1624 cm^- 1^, 1462⁓1425 cm^- 1^ and 875⁓874 cm^- 1^ for all P-Sources^45,46^. The strong bands observed at bands around 1121⁓1033 cm^- 1^ referred to the presence of the stretching vibration mode of phosphate group (PO_4_^3-^), while the bands around 600⁓ 572 cm^- 1^ indicated the existence of the bending mode of phosphate groups^47,48^. Additionally, the peak at 671 in SSP was associated to free carbonate as impurity compounds^49^. Moreover, the bands around 470 cm^- 1^ assigned to the stretching phosphate and sulphate form, which appeared to be stronger in PR^45,49^. The surface morphology of BC, BA, PR, and SSP was determined by Scanning Electron Microscopy (SEM) analysis, and the results obtained were provided in Fig. 2. In general, all different P-Sources displayed irregular and amorphous particles with a heterogeneous surface, and a small microporosity.

### Incubation experiment

#### Response surfaces

**Fig. 3:**
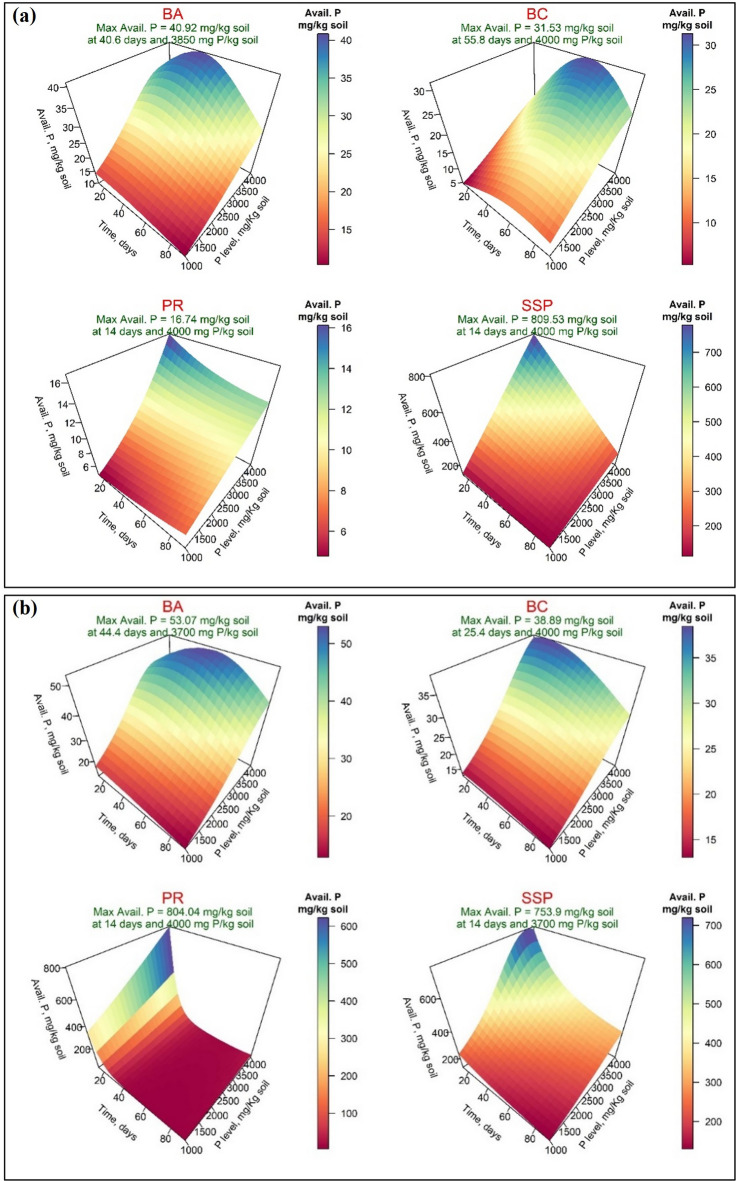
Response surface of available phosphorus (Avail. P, mg/kg) in calcareous soil amended with four phosphorus sources using distilled water (**a**) or acidified water (**b**): bone char (BA), bone ash (BC), phosphate rock (PR), and single superphosphate (SSP).

**Fig. 4:**
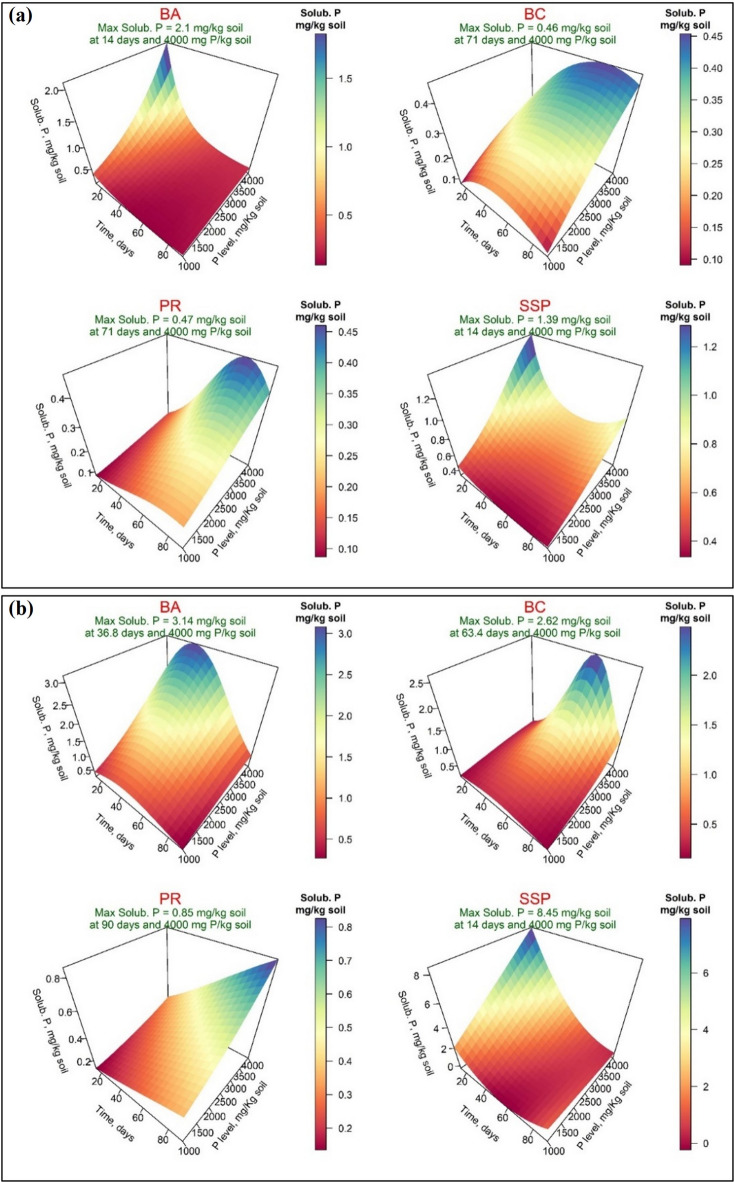
Response surface of soluble phosphorus (Avail. P, mg/kg) in calcareous soil amended with four phosphorus sources using distilled water (**a**) or acidified water (**b**): bone char (BA), bone ash (BC), phosphate rock (PR), and single superphosphate (SSP).

Figure 3 presented the response surfaces of the fitted model for available phosphorus (Avail. P, mg/kg) of the four P-Sources using distilled water (Fig. 3-a) and acidified water (Fig. 3-b). Under DW treatment, the highest maximum available P was observed for SSP (809.53 mg/kg), achieved after 14 days with a phosphorus application level of 4000 mg/kg. This was followed by BA (40.92 mg/kg) after 40.60 days at a phosphorus level of 3850 mg/kg. The maximum available P of bone char application was 31.53 mg/kg soil, achieved after 55.80 days at phosphorus level of 4000 mg P/kg, while PR exhibited the lowest value of the maximum available (16.74 mg/kg) after 14 days at 4000 mg P/kg. On the other hand, when acidified water (AW) was applied, the maximum available P values for the BA, BC, PR, and SSP were 53.07, 38.89, 804.04, and 753.9 mg/kg soil after 44.40, 25.40, 14, and 14 days, respectively. These levels were achieved at a P level of 3700 mg/kg for BA and SSP amended soil and 4000 mg/kg for BC and PR (Fig. 3-b). Concerning the soluble phosphorus (Solub. P, mg/kg) under distilled water treatment, Figs. 4-a and 4-b revealed that the maximum soluble P values application were 2.10, 0.46, 0.47, and 1.39 mg/kg soil for BA, BC, PR, and SSP, respectively, which were recorded after 14 days for BA and SSP and after 71 days for BC and PR, all at 4000 mg P/kg soil. When AW was applied, these values were increased to 3.14, 2.62, 0.85, and 8.45 mg/kg after 36.80, 63.40, 90, and 14 days, respectively, and at the addition of 4000 mg P/kg soil of the four P sources. Generally, the results of response variables revealed that the main effects of incubation time and P-level (PL) were significant for all four sources of phosphorus except the time effect for BC-amended soil on the available and soluble phosphorus when AW was applied and for PR-amended soil on available phosphorus when both wetting techniques were applied (Supplementary, Tables S-1 and S-2).

**Fig. 5:**
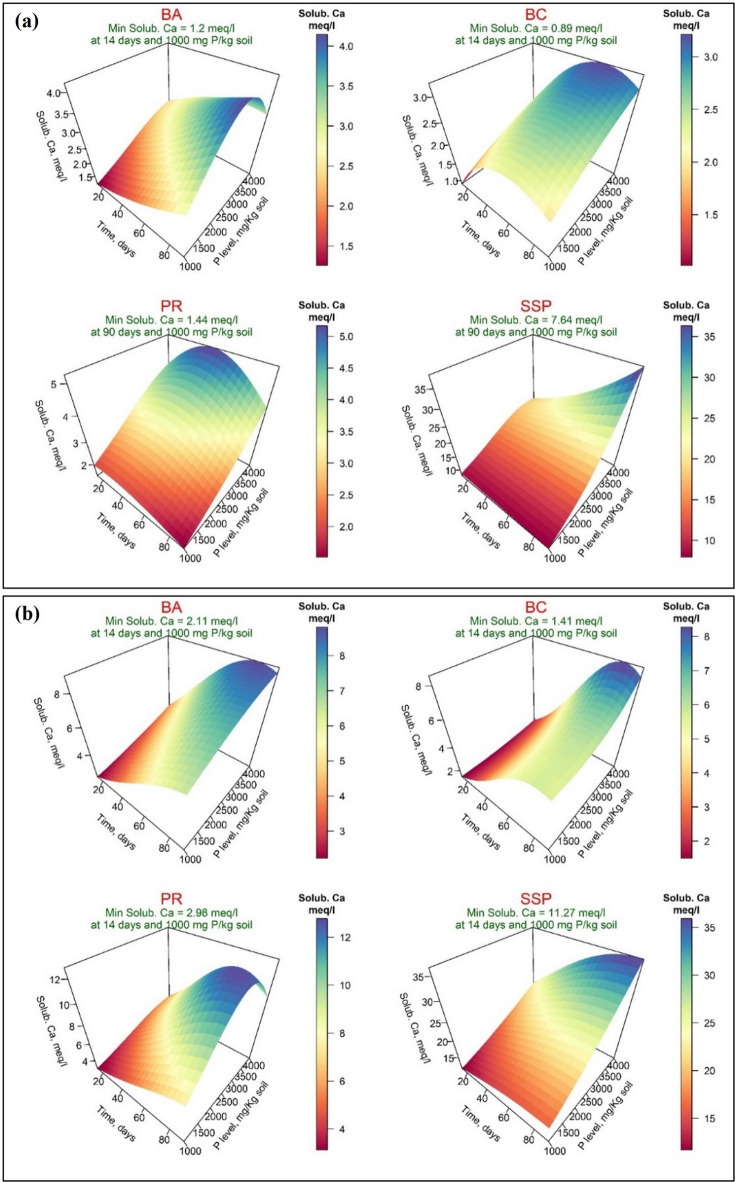
Response surface of soluble calcium (Solub. Ca^2+^, meq/l) in calcareous soil amended with different four phosphorus sources using distilled water (**a**) or acidified water (**b**): bone char (BA), bone ash (BC), phosphate rock (PR), and single superphosphate (SSP).

As displayed in Figs. 5-a and 5-b, the minimum soluble calcium (Solub. Ca^2+^, meq/l) values were 1.20, 0.89, 1.44, and 7.64 meq/l when DW was applied for BA, BC, PR, and SSP, respectively. However, when AW was applied, the values were 2.11, 1.41, 2.98, and 11.27 meq/l, respectively. The minimum values of soluble Ca^2+^, when DW was used, were recorded after 14 days for BA and BC and after 90 days for PR and SSP, all at a P level of 1000 mg P/kg soil. However, when AW was applied, the minimum values of soluble Ca^2+^ were significantly increased; these values were recorded after 14 days and at the addition of 1000 mg P/kg soil for all four P-Sources. Furthermore, the results related to response variables showed that the Incubation Time factor had a positive coefficient for BC and PR only when DW was used, while the PL factor had a positive coefficient that was observed for BC only when DW was used. The interaction was significant for BA when both wetting techniques were used and for PR only when AW was used. A negative coefficient for the interaction was detected for PR and SSP only when DW was used (Supplementary, Table S-3).

**Fig. 6:**
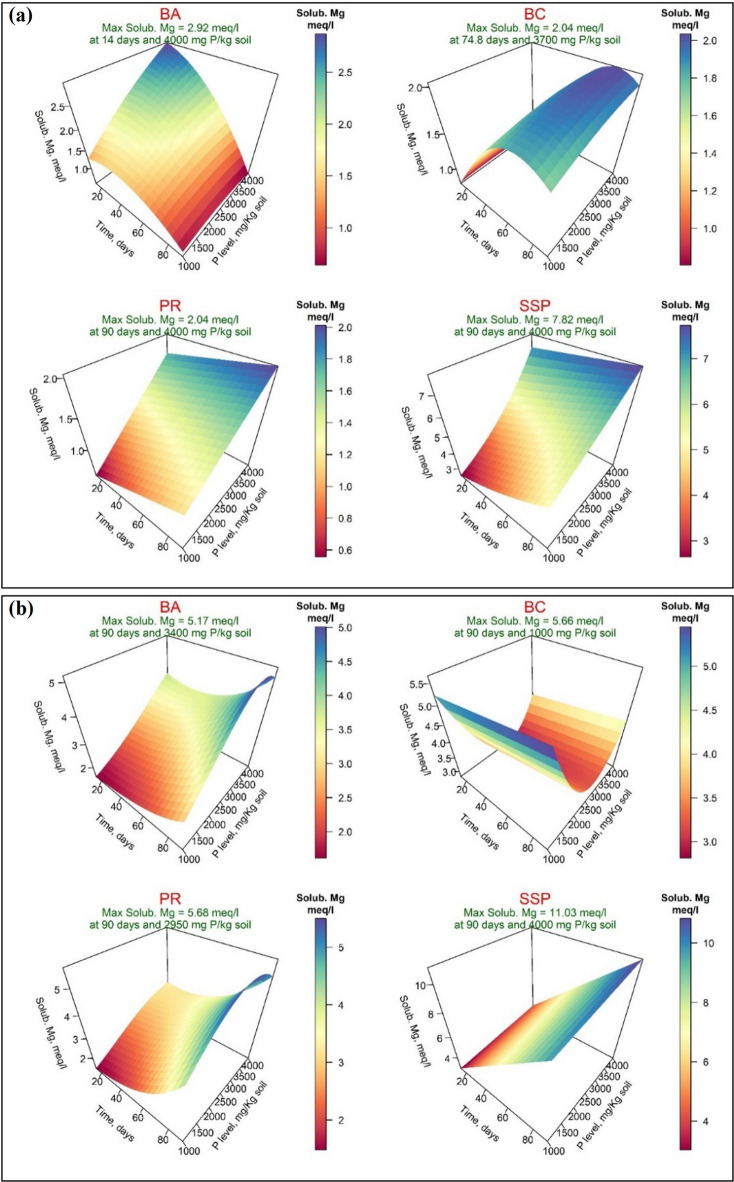
Response surface of soluble magnesium (Solub. Mg^2+^, meq/l) in calcareous soil amended with different four phosphorus sources using distilled water (**a**) or acidified water (**b**): bone char (BA), bone ash (BC), phosphate rock (PR), and single superphosphate (SSP).

The results of soluble magnesium (Solub. Mg^2+^, meq/l) are shown in Figs. 6-a and 6-b. The maximum values of Mg^2+^ were 2.92, 2.04, 2.04, and 7.82 meq/l for the four sources of P, respectively, when DW was used. While they were 5.17, 5.66, 5.68, and 11.03 meq/l for the BA, BC, PR, and SSP, respectively, when AW was used. These maximum values of Mg^2+^ were recorded after 14 and 74.8 days for BA, and BC, respectively, and after 90 days for PR and SSP and at the addition of 4000 mg P/kg soil for BA, PR, and SSP and at addition of 3700 mg P/kg soil of BC when DW was used. When AW was used, the maximum values of Mg^2+^ were recorded after 90 days for all the four sources of P and at the addition of 3400, 1000, 2950, and 4000 for BA, BC, PR, and SSP, respectively. The main effect of Incubation Time and PL factors was significant for all four sources of phosphorus except the BC when DW and AW were applied for the wetting process, while SSP showed insignificant when AW was used for PL factor only. The interaction was significant for BA when both wetting techniques were used and for PR when AW was used (Supplementary, Table S-4).

**Fig. 7:**
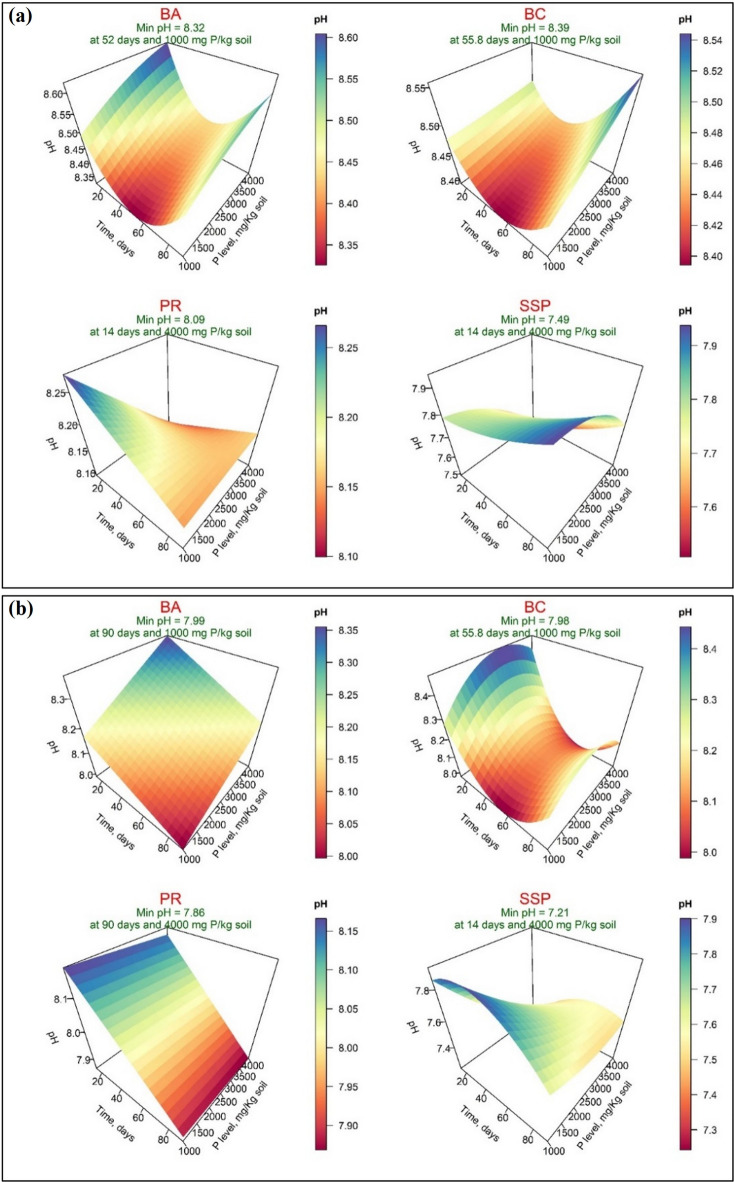
Response surface of pH in calcareous soil amended with four sources of phosphorus for applying distilled water (**a**) and acidified water (**b**): bone char (BA), bone ash (BC), phosphate rock (PR), and single superphosphate (SSP).

Concerning soil pH results, Figs. 7-a and 7-b showed that the minimum soil pH values were 8.32, 8.39, 8.09, and 7.49 when DW was used and were 7.99, 7.98, 7.86, and 7.21 when AW was used for BA, BC, PR, and SSP sources of P, respectively. When DW was used, the minimum values of soil pH were recorded after 52 and 55.8 days for BA and BC, respectively, and after 14 days for PR and SSP. These values were obtained at the P level of 1000 mg P/kg soil for BA and BC and 4000 mg P/kg soil for PR and SSP. While when AW was used, the minimum values of soil pH were recorded after 90 days for BA and PR, 55.8 days for BC, and 14 days for SSP, which was achieved at a P level of 1000 mg P/kg soil for BA and BC and at the addition of 4000 mg P/kg soil for PR and SSP. The linear effect of Time was not significant for BA, BC, and PR, when DW was used, except for SSP, which was significant. In contrast, the opposite trend was observed when DW was applied. However, the linear effect of PL was not significant for BC and PR only when AW was used. The interaction was significant for PR and SSP when distilled water and acidified water were used, respectively (Supplementary, Table S-5).

#### Resource’s optimization

The desirability function was used to optimize the five measured responses (available P, soluble P, soluble Ca^2+^, soluble Mg^2+^, and soil pH). The optimization aimed at maximizing available P, soluble P, and soluble Mg^2+^ simultaneously with minimizing soluble Ca^2+^ and soil pH. The importance (weight) of available P, soluble P, and pH was set to twice as soluble Ca^2+^ and soluble Mg^2+^ during the optimization process. The desirability function is given by Eq. (4):

4 $$\:{d}_{i}\left({\widehat{\mathbf{y}}}_{\mathbf{i}}\right)=\left\{\begin{array}{c}0,\:\:\:\:\:\:\:\:\:\:\:\:\:\:\:\:\:\:\:\:\:\:\:\:\:\:\:\:\:\:\:\:\:\:\:\:{\widehat{\mathbf{y}}}_{\mathbf{i}}\left(x\right)<\:{\mathbf{l}}_{\mathbf{i}}\\\:{\left(\frac{\:{\widehat{\mathbf{y}}}_{\mathbf{i}}\left(\mathbf{x}\right)-\:{\mathbf{l}}_{\mathbf{i}}}{{\mathbf{t}}_{\mathbf{i}}-{\mathbf{l}}_{\mathbf{i}}}\right)}^{{\varvec{\uptau\:}}_{1}},\:\:\:\:\:\:\:\:\:\:\:\:\:{\mathbf{l}}_{\mathbf{i}}\le\:{\widehat{\mathbf{y}}}_{\mathbf{i}}\left(x\right)\le\:\:{\mathbf{t}}_{\mathbf{i}}\\\:{\left(\frac{\:{\mathbf{u}}_{\mathbf{i}}\:-\:{\widehat{\mathbf{y}}}_{\mathbf{i}}\left(\mathbf{x}\right)\:}{{\mathbf{u}}_{\mathbf{i}}-{\mathbf{t}}_{\mathbf{i}}}\right)}^{{\varvec{\uptau\:}}_{2}},\:\:{\:\:\:\:\:\:\:\mathbf{t}}_{\mathbf{i}}\le\:{\widehat{\mathbf{y}}}_{\mathbf{i}}\left(x\right)\le\:\:{\mathbf{u}}_{\mathbf{i}}\\\:0,\:\:\:\:\:\:\:\:\:\:\:\:\:\:\:\:\:\:\:\:\:\:\:\:\:\:\:\:\:\:\:\:\:\:\:{\widehat{\mathbf{y}}}_{\mathbf{i}}\left(x\right)>\:{\mathbf{u}}_{\mathbf{i}}\end{array}\right.$$

*where* $\:{\tau\:}_{1}$and $\:{\tau\:}_{2}$are the weights that define the shape of the desirability function $\:{d}_{i}\left({\widehat{y}}_{i}\right)$, $\:{t}_{i}$, $\:{l}_{i},\:{\text{a}\text{n}\text{d}\:u}_{i}$ are the desired target, lower values, and upper values for the response variable, respectively.

So, if the aim is to maximize the value of the response, *y*_*i*_ for a given predictor variable *x*_*i*_, the desirability function *d*_*i*_(*y*_*i*_) is given by Eq. (5)

5 $$\:{\mathbf{d}}_{\mathbf{i}}\left({\widehat{\mathbf{y}}}_{\mathbf{i}}\right)=\left\{\begin{array}{c}0,\:\:\:\:\:\:\:\:\:\:\:\:\:\:\:\:\:\:\:\:\:\:\:\:\:\:\:\:\:\:\:\:\:\:\:\:{\widehat{\mathbf{y}}}_{\mathbf{i}}\left(x\right)<\:{\mathbf{l}}_{\mathbf{i}}\\\:{\left(\frac{\:{\widehat{\mathbf{y}}}_{\mathbf{i}}\left(\mathbf{x}\right)-\:{\mathbf{l}}_{\mathbf{i}}}{{\mathbf{t}}_{\mathbf{i}}-{\mathbf{l}}_{\mathbf{i}}}\right)}^{\varvec{\uptau\:}},\:\:{\mathbf{l}}_{\mathbf{i}}\le\:{\widehat{\mathbf{y}}}_{\mathbf{i}}\left(x\right)\le\:\:{\mathbf{t}}_{\mathbf{i}}\\\:1,\:\:\:\:\:\:\:\:\:\:\:\:\:\:\:\:\:\:\:\:\:\:\:\:\:\:\:\:\:\:\:\:\:\:\:{\widehat{\mathbf{y}}}_{\mathbf{i}}\left(x\right)>\:{\mathbf{t}}_{\mathbf{i}}\end{array}\right.\:\:$$

and if the aim is to minimize the value of the response, *y*_*i*_ for given predictor variables *x*_*i*_, the desirability function *d*_*i*_(*y*_*i*_) is given by

6 $$\:{\mathbf{d}}_{\mathbf{i}}\left({\widehat{\mathbf{y}}}_{\mathbf{i}}\right)=\left\{\begin{array}{c}1,\:\:\:\:\:\:\:\:\:\:\:\:\:\:\:\:\:\:\:\:\:\:\:\:\:\:\:\:\:\:\:\:\:{\widehat{\mathbf{y}}}_{\mathbf{i}}\left(\mathbf{x}\right)<{\mathbf{t}}_{\mathbf{i}}\\\:{\left(\frac{{\mathbf{u}}_{\mathbf{i}}-{\widehat{\mathbf{y}}}_{\mathbf{i}}\left(\mathbf{x}\right)}{{\mathbf{u}}_{\mathbf{i}}-{\mathbf{t}}_{\mathbf{i}}}\right)}^{{\varvec{\uptau\:}}_{2}},{\:\:\:\mathbf{t}}_{\mathbf{i}}\le\:{\widehat{\mathbf{y}}}_{\mathbf{i}}\left(\mathbf{x}\right)\le\:{\mathbf{u}}_{\mathbf{i}\:}\\\:0,\:\:\:\:\:\:\:\:\:\:\:\:\:\:\:\:\:\:\:\:\:\:\:\:\:\:\:\:\:\:\:\:{\widehat{\mathbf{y}}}_{\mathbf{i}}\left(\mathbf{x}\right)>{\mathbf{u}}_{\mathbf{i}}\end{array}\right.$$

the overall desirability function D is given by

7 $$\:\varvec{D}\:=\:{\left[\prod\:_{\varvec{i}=1}^{\varvec{c}}{\varvec{d}}_{\varvec{i}}\left({\widehat{\varvec{y}}}_{\varvec{i}}\right)\right]}^{1/\varvec{c}}=\:{\left[{\mathbf{d}}_{1}\left({\widehat{\mathbf{y}}}_{1}\right){\:\mathbf{d}}_{2}\left({\widehat{\mathbf{y}}}_{2}\right)...{\mathbf{d}}_{\mathbf{c}}\left({\widehat{\mathbf{y}}}_{\mathbf{c}}\right)\right]}^{1/\mathbf{c}}\:$$

where c is the number of responses.

**Fig. 8:**
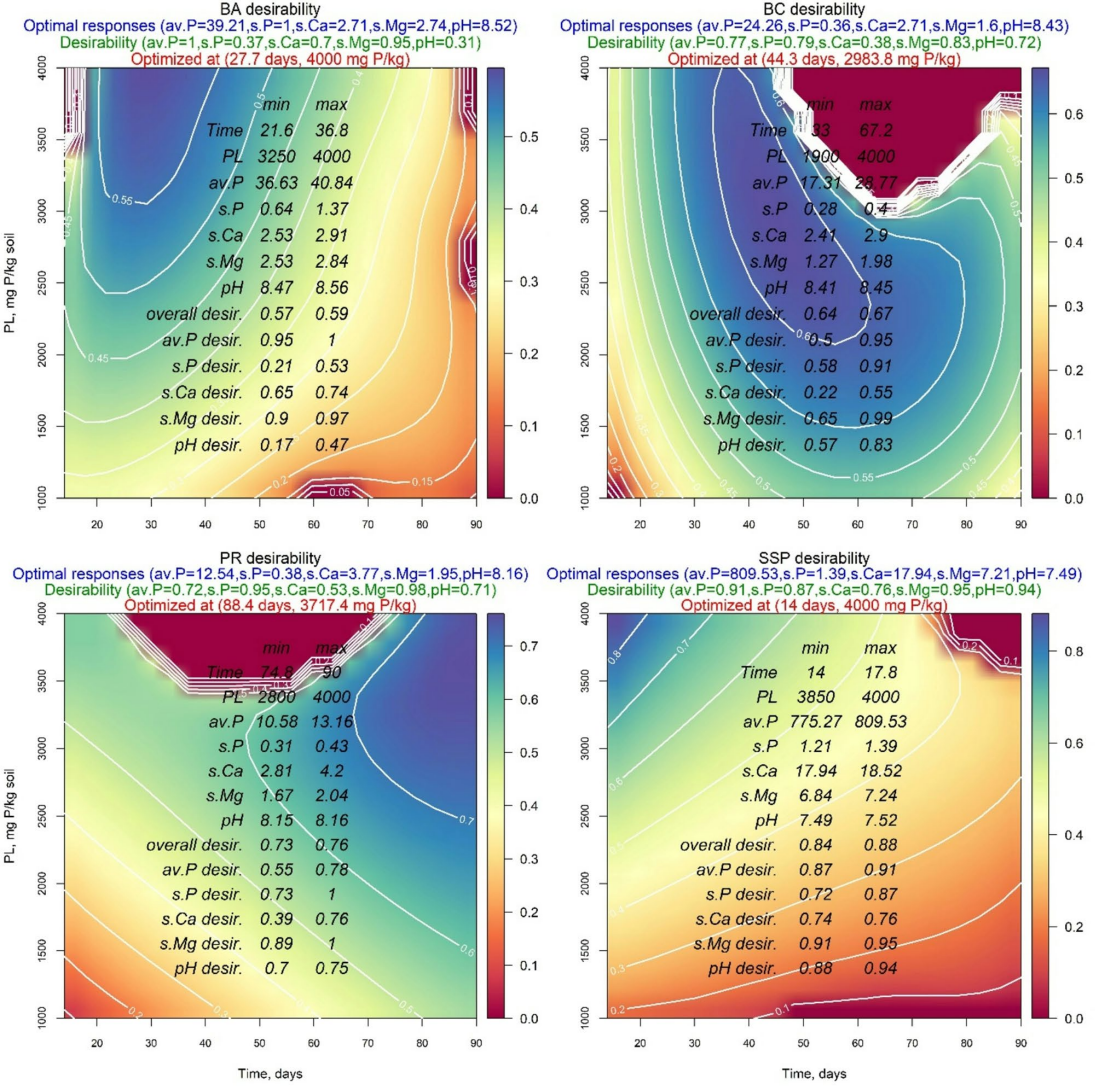
Contour plots of the four sources of phosphorus amendments in calcareous soil and wetted by distilled water (DW) showing optimal responses and their desirabilities as well as the optimized treatments: bone char (BA), bone ash (BC), phosphate rock (PR), and single superphosphate (SSP).

**Fig. 9:**
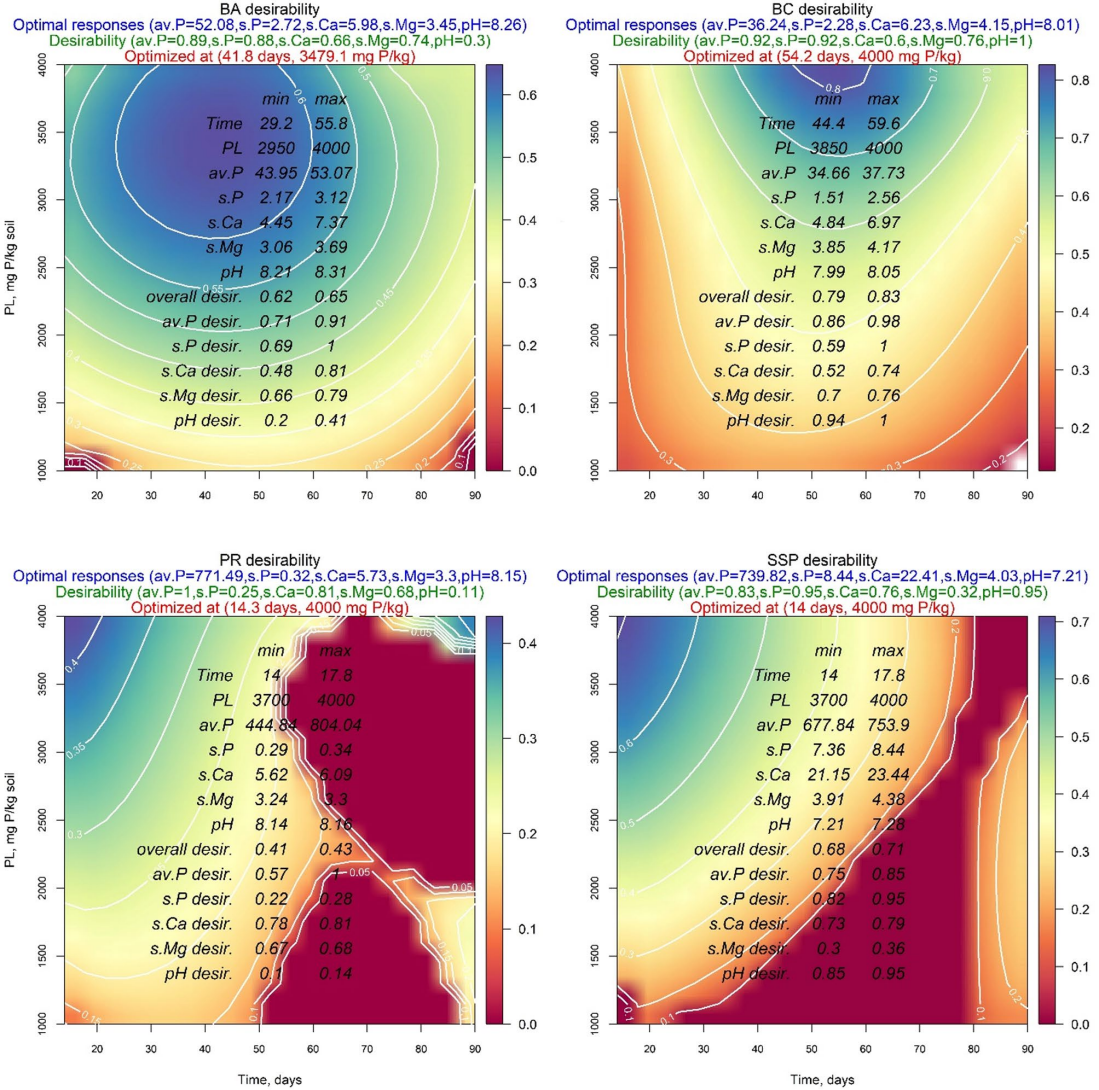
Contour plots of the four sources of phosphorus amendments in calcareous soil and wetted by distilled water (AW) showing optimal responses and their desirabilities as well as the optimized treatments: bone char (BA), bone ash (BC), phosphate rock (PR), and single superphosphate (SSP).

Figures 8 and 9 show that the maximum overall desirability of BA was 0.59 and 0.65 for applying DW and AW, respectively. However, the individual desirability values of applying DW were 1.00, 0.37, 0.7, 0.95, and 0.31 for available P, soluble P, soluble Ca^2+^, soluble Mg^2+^, and soil pH, respectively. While for acidified water application, these values were 0.89, 0.88, 0.66, 0.74, and 0.30, respectively. The optimal response values of the investigated parameters were 39.21 mg P/kg soil, 1.00 mg P/kg soil, 2.71 meq/l, 2.74 meq/l, and 8.52 when DW was used, and 52.08 mg P/kg soil, 2.72 mg P/kg soil, 5.98 meq/l, 3.45 meq/l, and 8.26 when AW was used for available P, soluble P, soluble Ca^2+^, soluble Mg^2+^, and soil pH, respectively. These optimal responses were obtained after 27.7 and 41.8 days at the addition of 4000 mg P/kg 3479.1 mg P/kg soil when DW and AW were applied, respectively (Table 4).

**Table 4:** Optimal predictors and responses for the four sources of phosphorus and two wetting techniques.

P-Source	Extraction method	Optimal predictors		Optimal responses					Individual desirabilities				
		Time, days	PL, mg kg	Avail. P, mg/kg	Solub. P, mg/kg	Solub. Ca, meq/l	Solub. Mg, meq/l	pH	Avail. P, mg/kg	Solub. P, mg/kg	Solub. Ca, meq/l	Solub. Mg, meq/l	pH
BA	DW	27.7	4000	39.21	1.00	2.71	2.74	8.52	1.00	0.37	0.70	0.95	0.31
	AW	41.8	3479.1	52.08	2.72	5.98	3.45	8.26	0.89	0.88	0.66	0.74	0.30
BC	DW	44.3	2983.8	24.26	0.36	2.71	1.60	8.43	0.77	0.79	0.38	0.83	0.72
	AW	54.2	4000	36.24	2.28	6.23	4.15	8.01	0.92	0.92	0.60	0.76	1.00
PR	DW	88.4	3717.4	12.54	0.38	3.77	1.95	8.16	0.72	0.95	0.53	0.98	0.71
	AW	14.3	4000	771.49	0.32	5.73	3.30	8.15	1.00	0.25	0.81	0.68	0.11
SSP	DW	14	4000	809.53	1.39	17.94	7.21	7.49	0.91	0.87	0.76	0.95	0.94
	AW	14	4000	739.82	8.44	22.41	4.03	7.21	0.83	0.95	0.76	0.32	0.95

BC: bone char; BA: bone ash; PR: phosphate rock; and SSP: single superphosphate.

DW: distilled water; AW: acidified water; PL: phosphorus level.

Avail. P: available phosphorus; Solub. P: soluble phosphorus; Solub. Ca: soluble calcium; Solub. Mg: soluble magnesium.

Additionally, the same Figures and Table show that BC maximum overall desirability was 0.67 and 0.83 for applying DW and AW, respectively. However, the individual desirability values were 0.77, 0.79, 0.38, 0.83, and 0.72 when DW was used and 0.92, 0.92, 0.60, 0.76, and 1.00 when AW was used for available P, soluble P, soluble Ca^2+^, soluble Mg^2+^, and soil pH, respectively. The optimal response values were 24.26 mg P/kg soil, 0.36 mg P/kg soil, 2.71 meq/l, 1.60 meq/l, and 8.43 when DW was used and were 36.24 mg P/kg soil, 2.28 mg P/kg soil, 6.23 meq/l, 4.15 meq/l, and 8.01 when AW was used for the same investigated parameters, respectively. These optimal responses due to DW application were obtained after 44.3 days and the addition of 2983.8 mg P/kg soil, while those for applying AW were obtained after 54.2 days and at the addition of 4000 mg P/kg soil (Table 4).

Concerning PR, the value of maximum overall desirability was 0.76 when DW was used and 0.43 when AW was used. However, the individual desirability values were 0.72, 0.95, 0.53, 0.98, and 0.71 when DW was applied and 1.00, 0.25, 0.81, 0.68, and 0.11 when AW was applied for available P, soluble P, soluble Ca^2+^, soluble Mg^2+^, and soil pH respectively. The optimal responses values were 12.54 mg P/kg soil, 0.38 mg P/kg soil, 3.77 meq/l, 1.95 meq/l, and 8.16 when DW was used and were 771.49 mg P/kg soil, 0.32 mg P/kg soil, 5.73 meq/l, 3.30 meq/l, 8.15 when AW was used for available P, soluble P, soluble Ca^2+^, soluble Mg^2+^, and soil pH, respectively. These optimal responses were obtained after 88.4 days and at the addition of 3717.4 mg P/kg soil when DW was used and were obtained after 14.3 days and at the addition of 4000 mg P/kg soil when AW was used (Table 4).

The results for SSP demonstrated the highest values of the overall desirability compared to the other P-Sources. Specifically, the individual desirability values were 0.91, 0.87, 0.76, 0.95, and 0.94 when DW was used and were 0.83, 0.95, 0.76, 0.32, and 0.95 when AW was used for available P, soluble P, soluble Ca^2+^, soluble Mg^2+^, and soil pH, respectively. Furthermore, the optimal response values showed the highest values across all investigated parameters, except for the soil pH value, which was the lowest among studied P-Sources (Table 4). These optimal responses were achieved after 14 days and with the addition of 4000 mg P/kg soil when applying DW or AW.

**Fig. 10:**
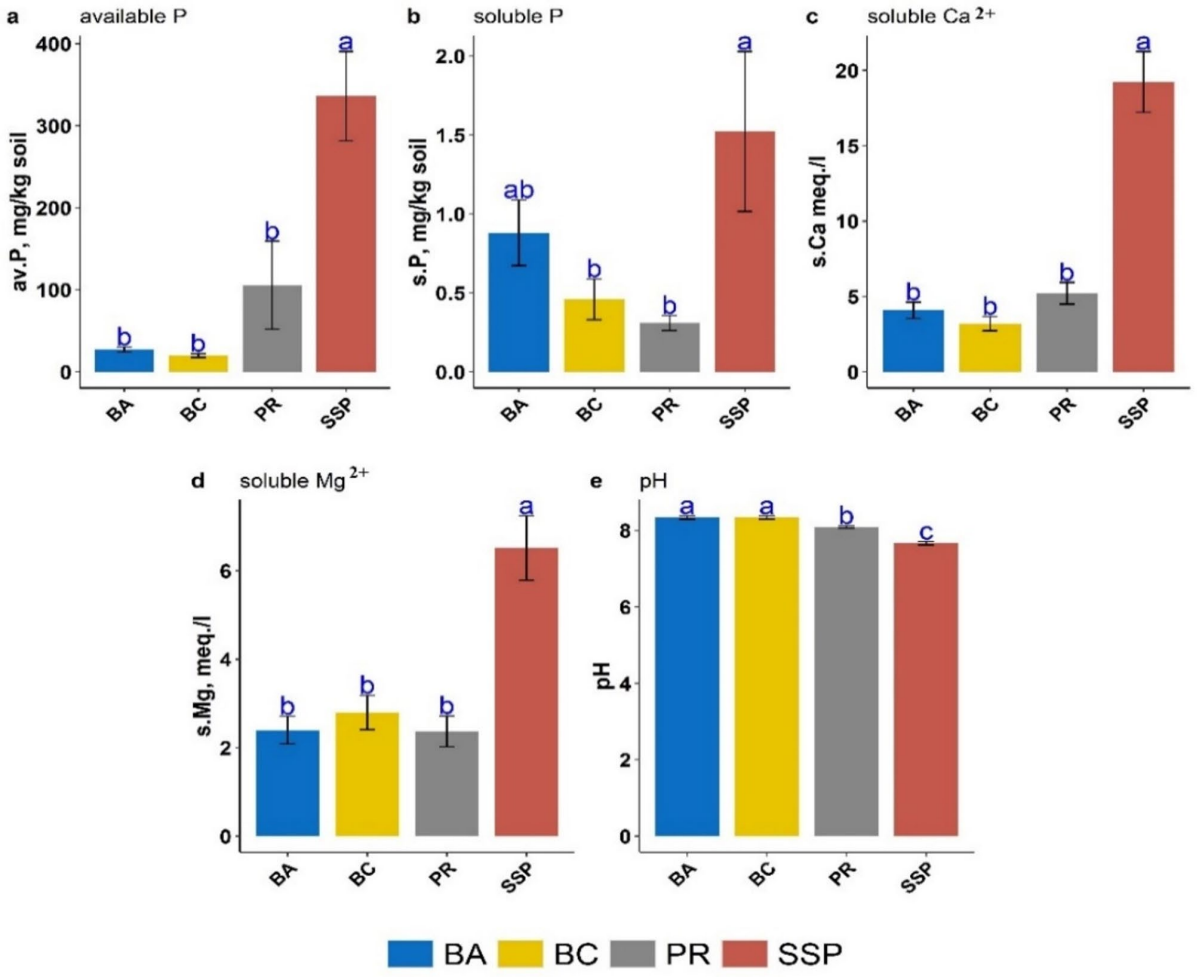
Main effect of the four sources of phosphorus based on Tukey multiple comparison test (interaction was not significant): bone char (BA), bone ash (BC), phosphate rock (PR), and single superphosphate (SSP).

**Fig. 11:**
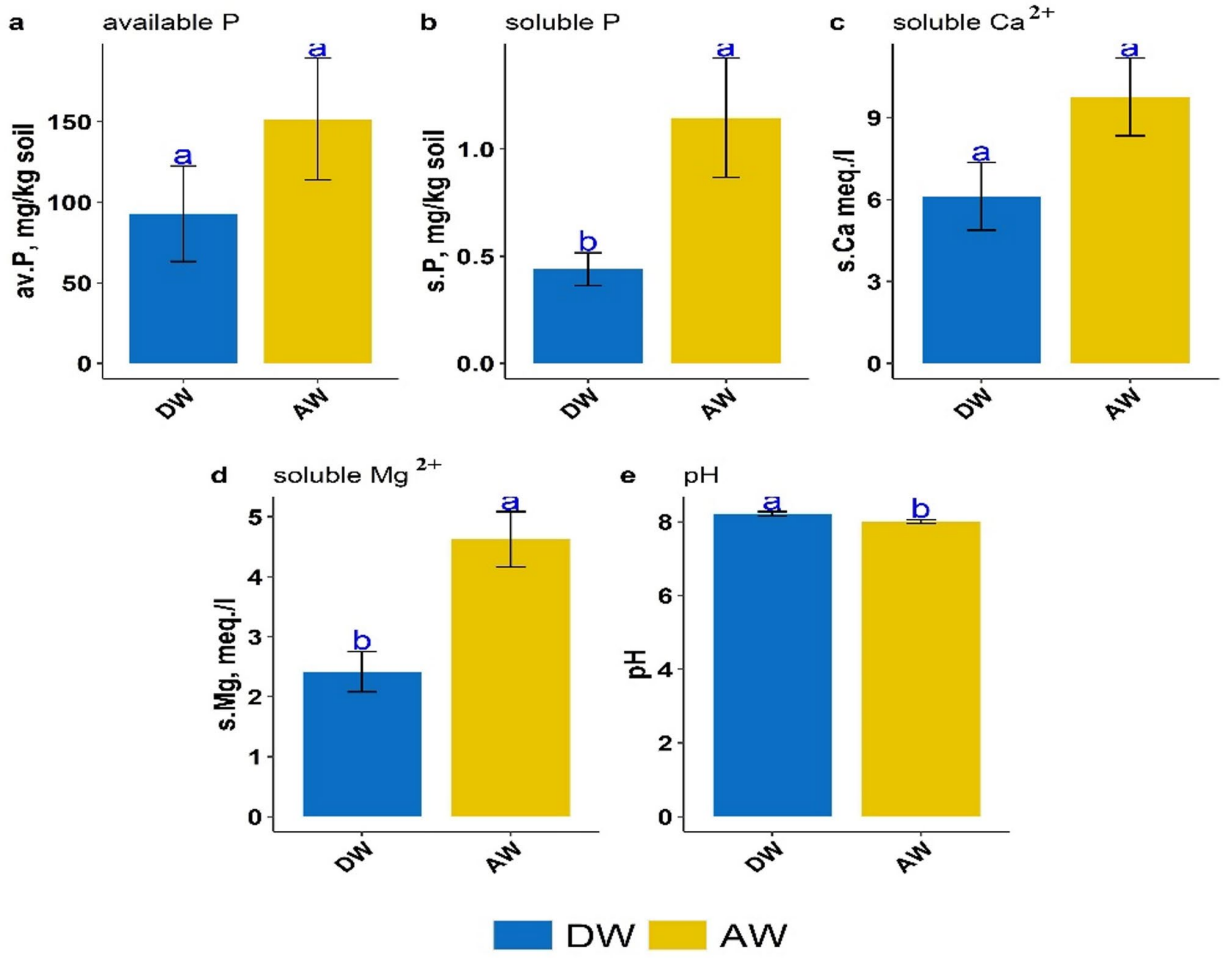
Main effect of wetting type based on Tukey multiple comparison test (interaction was not significant): distilled water (DW) and acidified water (AW).

Figure 10 showed the comparison among the four sources of P according to Tukey multiple comparison test. Figure 10 (a, c, d, and e) revealed that SSP was significantly different from the other 3 sources of P with respect to available P, soluble (Ca^2+^ and Mg^2+^), and pH, respectively. Figure 10-b revealed that there was no significant difference between SSP and BA with respect to soluble P. In the same context, Fig. 11 demonstrated the comparison between the two methods of extraction (AW and DW) where there was no significant difference between them with respect to available P and soluble Ca^2+^ (Fig. 11-a and 11-c). However, AW was superior and significantly different from DW for both soluble P and soluble Mg^2+^ (Fig. 11-b and 11-d). Figure 11-e revealed that despite the very small difference between AW and DW in pH, it was significant.

## Discussions

The P_2_O_5_ concentration in the bone char (BC), bone ash (BA), phosphate rock (PR), and single superphosphate (SSP) may vary due to differences in their source materials, formation processes, and overall composition. It was concluded that the lower the Ca/P molar ratio, the greater the solubility and mobility of phosphate in soil^50^. Small variations in the Ca/P ratio can lead to significant differences in phosphorus solubility, as this ratio influences the composition, crystallinity, and stability of P-containing mineral phases. It was reported that when the Ca/P ratio is below 1.5, the phosphorus fraction becomes more soluble (labile). Conversely, when the ratio exceeds 1.67, phosphorus tends to be bound in recalcitrant mineral structures, resulting in reduced solubility and increased fixation in the soil^51^. Interestingly, the levels of heavy metals detected in the bone char, bone ash, phosphate rock, and single superphosphate were generally below permissible limits regardless of the cadmium in the PR and SSP, which exceeded the maximum allowable concentration. However, phosphate rocks and derived fertilizers are known to be potential sources of heavy metals contamination, particularly cadmium and lead. Notably, it is reported that the maximum permissible levels of heavy metals in soil are 3.00 mg kg^- 1^ for cadmium, 100.00 mg kg^- 1^ for copper, 50,000 mg kg^- 1^ for iron, 2,000 mg kg^- 1^ for magnesium, and 100 mg kg^- 1^ for lead^52^. The accumulation and bioavailability of these metals in soil, subsequently uptake by grown plants, depend not only on their initial concentration in the commercial fertilizers but also on the rates of application, biological activity, and soil chemical properties, such as soil pH, cation exchange capacity (CEC), and organic matter content and its type, that are influenced by long-term application of P fertilizers^53^. Thus, using bone-based materials as alternative phosphorus fertilizers is a promising strategy to avoid contamination with heavy metals^8^. Bone char exhibited the highest carbon, which contributes to improved surface properties and porosity of the material. Moreover, the presence of carbon in bone char enhances phosphorus availability by modifying adsorption/desorption dynamics and supporting microbial processes that mediate P cycling and solubilization in soils^51,54^.

The X-ray diffraction patterns of different P-Sources confirmed the presence of hydroxyapatite [Ca_10_(PO_4_)_6_(OH)_2_] and calcium carbonate CaCO_3_, indicating the formation of calcite structure formation^22,48,55^. The XRD patterns of bone ash are sharper than those of bone char; this may be because BC has higher organic carbon than that detected by BA (Table 3), which is associated with the bone char structure. The presence of organic carbon may interrupt the formation of apatite crystals during the pyrolysis process. Moreover, the crystalline phase of gypsum [Ca(SO_4_).2H_2_O], which forms during the manufacturing of P-fertilizer, was confirmed by the relatively high concentration of sulphur in single superphosphate (Table 3) compared to the other P-Sources^49^. Furthermore, the OH group detected by infrared spectra in SSP and BA verifies the presence of hydroxyapatite in bone ash composition^56,57^. The peak at 2355 cm^- 1^ in BC has disappeared in BA, which may be interpreted as the elimination of fixed carbon during the thermo-combustion process, where it is released as volatile carbon gases^56^. Additionally, the presence of phosphate group (PO_4_^3-^) peaks indicates the characteristic of the apatite crystal form^44,58^. Several studies have reported that the FT-IR spectrum of bone ash closely resembles that of hydroxyapatite, which is confirmed by the presence of PO_4_^3-^, –OH, and CO_3_^2-^ bonds^44,56–58^. The major components of SSP are monocalcium phosphate and gypsum, which were confirmed by the presence of vibration bands corresponding to OH^–^, SO_4_^2-^, and PO_4_^3–49^. The rugged structural characteristics of BC detected by SEM indicate the successful removal of organic residues (meat, fat, and collagen) from cattle bone structure during the pyrolysis process (Fig. 2-BC). It is well known that the main components of BC are fixed carbon and hydroxyapatite^45^. Additionally, the bright regions observed in Fig. 2-BA indicate the formation of hydroxyapatite during the thermo-combusting process. Thermal treatment of bone above 600 eliminates organic matter from the bone and augments the crystallinity of hydroxyapatite^59^.

The availability and solubility of phosphorus from various studied P-Sources in the incubated calcareous soil exhibited variability. These variations depend on the differences in sources, phosphorus level, incubation time, and wetting techniques (distilled vs. acidified water). In general, incubation periods significantly increased the content of available P in calcareous soil compared to the original soil level of 5 mg/kg (Table 1). Irrespective of single superphosphate fertilizer (SSP), the overall mean response of bone ash and bone char to phosphorus solubility and availability was significantly positive when distilled water was used for wetting applications. The maximum amount of available phosphorus released during the incubation time was obtained from bone ash (40.32 mg P/kg), followed by bone char (31.53 mg P/kg), and then phosphate rock (16.74 mg P/kg). In contrast, when acidified water was applied, the solubility rate followed the order PR > BA > BC. According to Saleh et al.^8^, the solubility and availability of phosphorus from bone ash and bone char (non-traditional P-Sources), as well as phosphate rock (traditional sources) using different extraction techniques, bone ash exhibited the highest phosphorus solubility, followed by bone char pyrolyzed at 600 °C, while phosphate rock had the lowest solubility^8^. Interestingly, the lower phosphorus solubility and availability observed in BC-amended soil compared to BA-amended soil may be attributed to the presence of organic compounds that form less labile organic phosphorus. This result was demonstrated by the XRD pattern shown in Fig. 1-a, which confirmed that the phosphorus content in bone char may be sustained in soil and become more available under planting conditions for several agricultural seasons^60^. In contrast, PR released phosphorus more slowly due to its higher alkalinity; additionally, it is scarcely soluble under alkaline conditions but highly soluble in acidic environments^8^. From another point of view, single superphosphate demonstrated the highest phosphorus availability and solubility due to its lowest ratio of Ca/P (1.38, Table 3), followed by bone char (1.68) and bone ash (1.73).

As a result, bone char and bone ash appear to be valuable resources for P recovery in agricultural systems. It was reviewed that phosphorus dynamics in soil depend on various factors, including particle size distribution, soil structure, soil pH, redox reactions, organic matter content, soil salinity, major minerals composition of soil, leachate and runoff, soluble and total phosphorus content, soil enzymes activity, presence of phosphate-dissolving bacteria, and mycorrhizas^15,61^. Among these, soil pH is the primary influence on the quantities of phosphorus released from various P-Sources. Herein, soil pH decreased with incubation time even when applying distilled water or acidified water. The lowest values of soil pH were observed for SSP application, which interprets its higher phosphorus solubility compared to the other three P-Sources (Fig. 10). However, Do Nascimento et al.^62^ studied the behavior and mobility of phosphorus from three different phosphorus sources (calcium, magnesium, and ammonium) in Petri dishes incubated soil over 56 days using soil from the United States (Mollisol; pH 8.0) and Brazil (Ultisol; pH 6.6). They observed that calcium phosphate (TSP) was superior to decreasing soil pH under all tested conditions, especially in alkaline soil with an initial pH of 8.0. Alongside the acidic properties of this source and the displacement of H^+^ ions from the cation exchange capacity into the soil solution due to the rise in Ca concentration, indicating that phosphate ions exhibit three protonation constants, with the dominant forms are H_2_PO_4_^-^, HPO_4_^2-^, and PO_4_^3-^ at pH 2.1, 7.2, and 12.6, respectively. Thus, phosphate ions tend to deprotonate when applied to soils with a pH exceeding 7.2, subsequently reducing soil pH due to donating H^+^ ions to the soil solution^63^. The minimum soil pH values ranged from 7.49 to 8.39 with distilled water and from 7.21 to 7.99 with acidified water across the four sources. This confirmed that the dominant form is mono-hydrogen phosphate (HPO₄²⁻). Additionally, the decrease in soil pH led to increased solubility of calcium and magnesium in soil amended with different P-Sources, which significantly affects the solubility and availability of phosphorus. However, these results are confirmed in Figs. 10 and 11. The same results were confirmed by Saleh et al.^8^, who reported that the pH of incubated sandy calcareous soil amended by bone char (pyrolyzed at 600 °C), bone ash, phosphate rock, and single superphosphate in the presence of N-K fertilizers decreased over 120 days (≃29 °C) by an average of 0.2, 0.17, 0.1, and 0.79 units relative to unamended soil, respectively^8^. The soil pH decrease led to an increase in calcium solubility and subsequently in the availability of phosphorus; this may be due to the dissolution of calcium carbonate^64^. Notably, phosphate transforms into the more basic forms, and protons (H^+^) are released, providing carbonate solubility and accelerating calcium dissolution^65^. By optimizing soil Ca^2+^ and Mg^2+^ solubility alongside soil pH management, untraditional and alternative phosphorus sources, including bone char and bone ash, can be effectively utilized as slow-release phosphorus fertilizers. Forms and transformations of phosphorus in soils are influenced by a combination of several factors, such as soil properties, P sources characterization (inorganic and organic fertilizers), agricultural practices, and hydrology conditions. These factors effectively control the phosphorus solubility and availability in soil and significantly play a vital role in maintaining the long-term phosphorus fertility of the soil system. It is important to note that bone char or bone ash, as low-released fertilizers, may not supply sufficient phosphorus for optimal plant growth in the short term. Therefore, higher application rates may be necessary to achieve the required concentrations.

## Conclusion

Bone char showed the highest fundamental element components (C, H, and N), as well as the greatest surface area and total pore volume among the phosphorus sources examined. In contrast, bone ash exhibited the highest values for both the Ca/P ratio and mean pore diameter. Notably, the Ca/P ratio varied among the studied P-Sources, which has a prevalent effect on phosphorus release behavior in soil. The solubility and availability of phosphorus from the studied P-Sources in the incubated calcareous soil were significantly affected by phosphorus level, incubation time, and wetting techniques (distilled vs. acidified water). Single superphosphate consistently demonstrated the highest phosphorus solubility and availability due to its relatively low Ca/P ratio. Regardless of the behavior of single superphosphate, both bone-based materials exhibited significant increases in soluble and available phosphorus when distilled water was applied. The highest amount of phosphorus release was observed for bone ash, followed by bone char, and the lowest was phosphate rock. In comparison with acidified water, phosphate rock resulted in a higher release rate, followed by bone ash and bone char. This is primarily due to a reduction in soil pH. This study confirmed that the soil pH reduction over incubation time, regardless of the wetting techniques, plays a crucial role in P-dynamics in calcareous soil, with lower pH values promoting the solubility of calcium and magnesium, leading to greater P solubility and availability in soil. This reflected the dominant formation of mono-hydrogen phosphate (HPO₄²⁻) in calcareous soil. The wetting technique significantly affects the optimal incubation time (day) and phosphorus levels (mg P/kg soil). Furthermore, the optimal response for available and soluble phosphorus in calcareous soil with acidified water was higher than distilled water for all tested P-Sources. Bone char and bone ash are eco-friendly and sustainable resources for phosphorus fertilizers in agricultural systems due to their potential to enhance soil properties and soil fertility over time. Interestingly, the selection of phosphorus fertilizer depends on its physicochemical characterizations, soil properties, and management practices to optimize the solubility and availability of phosphorus for plant growth requirements. Overall, the results obtained from this study illustrated the importance of P-Source selection based on management practices to optimize phosphorus solubility and availability in order to meet the requirement of phosphorus fertilizers for enhancing plant growth. Future studies are necessary to assess the long-term effects of bone char and bone ash under field conditions and their interactions with other soil amendments and fertilizers to further enhance their agricultural applications and soil fertility management.

## Supplementary Information

Below is the link to the electronic supplementary material.

Supplementary Material 1

## Data Availability

All data generated or analyzed during this study are included in this published article (andits supplementary information file).
