# Supplementary material for: Optimizing phosphorus released in calcareous soil amended with bone char and bone ash using response surface methodology and desirability function
Source: Sci Rep. 2025 Aug 18;15:30198. doi: 10.1038/s41598-025-13548-5 (PMC12361385; doi:10.1038/s41598-025-13548-5)
Supplement: Supplementary file 1 — Supplementary Material 1 [file 41598_2025_13548_MOESM1_ESM.docx]

**Optimizing Phosphorus Released in Calcareous Soil Amended with Bone Char and Bone Ash Using Response Surface Methodology and Desirability Function**

Yasser A. El-Damarawy^1^, Eman M. Saleh^2^, Omar M. Ibrahim^3^, Ahmed El‑Refaey^4^ Maher E. Saleh^2*^, Eman H. El-Gamal^5*^

## **Fitted model**

The coefﬁcients ± standard error for linear, two-way interaction, and quadratic terms of the full quadratic polynomial model are summarized in [Tables S-1](#_bookmark3) through S-5. The model goodness of fit and predictive performance was evaluated by 7 indicators, R^2^, adjusted R^2^, predictive R^2^, normalized root mean squared error (nRMSE), lack of fit, normality of the response, and normality of the residuals. A good model should have higher values of R^2^, adjusted R^2^, and predictive R^2^ with a difference no more than 0.2 between adjusted R^2^ and predictive R^2^ in most cases where high values of R^2^ and adjusted R^2^ suggest that the model fits the data well and captures a large portion of the variation in the response. While predictive R^2^ metric assesses the model's ability to predict new observations. High value of predictive R^2^ with a difference no more than 0.2 from adjusted R^2^ indicates that the model has good predictive accuracy. The normalized root mean squared error represents the average deviation between observed and predicted values, expressed as a percentage of the mean response. A value less than 20% suggests that the model has a relatively low error rate. The lack-of-fit test assesses whether a model adequately fits the data. A good model should have insignificant lack-of-fit (*P* > 0.05). Sometimes fitting the model to the data needs to transform the response variable by different methods as illustrated in [Tables S-1](#_bookmark3) through S-5.

*Response variables*

A positive effect means that a direct proportional relationship existed, where negative effect indicates inverse proportional relationship. The negative coefficient of the interaction term suggests that when the time increased, the effect of PL on the response decreased and vice versa. In other words, the combined effect of both incubation time and PL is less than the sum of their individual effects indicating antagonism behavior. On the other hand, when an interaction coefficient is positive, this suggests that when the time increased, the effect of PL on the response increased. This means the combined effect of both incubation time and PL is more than the sum of their individual effects indicating synergistic behavior. The role of quadratic effect is to capture the curvature in the response surface, and consequently allowing for a better representation of the relationship between the treatments and the response.

*Available phosphorus*

The main effects of the incubation time and phosphorus levels (PL) factors of the studied P-Sours have significant effects on the availability of phosphorus except the time effect for PR- and BC-amended soil when DW and AW were applied for wetting cycles, respectively (Table S-1). Generally, the main effect of time was positive on the availability of phosphorus, except for SSP- and PR-amended soil when DW and AW were applied, respectively. With respect to the main effect of PL, the positive coefficient was observed when DW was applied except for BA-amended soil, where it was negative. On the other hand, when the acidified water was applied, the PL coefficient was negative except for PR-amended soil. Concerning the interaction between the time and PL captures their effect on phosphorus availability; It was significant when DW was used for wetting process except for BC. When AW was used, it was insignificant except for BA. Generally, the interaction term was negative except for BC- and PR-amended soils under AW application. The squared terms represented the quadratic effects of the incubation time and PL factors on available phosphorus. Both quadratic terms were statistically significant in BA- and BC-amended soil with DW application, suggesting nonlinear relationships between these predictors and the response. On the other hand, they were not significant in PR when DW was applied for wetting cycles. For SSP-amended soil with DW application, the fitted model was linear, so no quadratic effects existed. Both Time^2^ and PL^2^ were significant when AW was applied in BA-amended soil. However, in BC- and SSP-amended soils, only PL^2^ was significant, while in PR, only the Time^2^ of incubation was significant.

*Soluble phosphorus*

The results in Table S-2 revealed that the main effect of incubation time and PL were significant for all the four sources of phosphorus except for BC when AW was used for the time only. With respect to the time factor, a positive coefficient was observed for BC and SSP when DW was applied and for PR when AW was used for wetting cycles. While for PL, the negative coefficient was observed for PR and SSP when DW was used and for BC when AW was used. The interaction between the incubation time and PL was significant for BC and for BA and SSP when DW and AW were used, respectively. The interaction coefficient was positive for BC and PR with DW application. Time^2^ was significant for all P-Sources except for PR when AW was used. While PL^2^ was significant for BC and SSP only when DW was applied. The coefficient of Time^2^ was negative for BC and SSP and for BA and PR when DW and AW was used, respectively. The coefficient of PL ^2^ was negative for BA and BC only when DW was used and for BA only when AW was used.

*Soluble Calcium*

The main effect of the incubation time and PL on calcium solubility were significant for all the four sources of phosphorus except for PR and SSP when DW was used for time only (Table S-3). A positive coefficient related to the incubation time factor was observed for BC and PR only with DW application, while in PL factor the positive coefficient was observed for BC only when DW was used. The interaction was significant for BA when DW was used and for BA and PR only when AW was used. A negative coefficient for the interaction was detected for PR and SSP only when DW was used. The quadratic effect of Time^2^ was significant for all the four P-Sources except for SSP when DW was used. While the quadratic effect of PL^2^ was insignificant for all the four sources of P except for BA and SSP when DW was used and for PR when AW was used.

*Soluble Magnesium*

For soluble magnesium, a quadratic model was fitted for BA and BC with both DW and AW for the witting process, while PR exhibited more fit for this model only when AW was applied. In contrast, a better fit of the linear model was observed for PR and SSP with the DW application and for SSP with the AW application. The results in Table S-4 explained that the main effect of incubation time and PL on the soluble Mg from the different P-Sources application based on type of water used for wetting incubation periods. It appeared that the time factor had a significant effect on the solubility of Mg for all P-Sources applications except for BC with AW application. Additionally, the PL factor was significant, expect for BC with DW and SSP with AW. Regarding the interactions, significant effects were observed for BA only when DW was used and for BA and PR with AW. Furthermore, the quadratic effect of Time^2^ was significant for BA and BC when DW was applied and for PR with AW. The quadratic effect of PL^2^ was significant for BA, BC, and PR when AW was used.

*Soil pH*

A linear model was fitted for PR when DW was used and for BA and PR when AW was used. While a quadratic model was fitted for the rest. The linear effect of time was not significant for BA, BC, and PR when DW was used and for SSP when AW was used. While the linear effect of PL was not significant for BC and PR only when AW was used. The interaction was significant for PR when DW was used and for SSP when AW was used. The quadratic effect of Time^2^ was significant for the P sources that fitted with quadratic model except for SSP when DW was used. Also, the quadratic effect of PL^2^ was significant for SSP only when DW was used (Table S-5).

|  |  | BA | BC | PR | SSP |
| --- | --- | --- | --- | --- | --- |
| Intercept | DW | 0.04±0.002^***^ | 1.35±0.021^***^ | 0.96±0.028^***^ | 313.49±17.545^***^ |
|  | AW | 0.03±0.001^***^ | 0.04±0.002^***^ | 1.08±0.029^***^ | 0.003±0.0002^***^ |
| Time | DW | 0.01±0.001^***^ | 0.11±0.016^**^ | 0.02±0.022^ns^ | -153.51±23.756^***^ |
|  | AW | 0.006±0.0008^***^ | 0.004±0.002^ns^ | -0.76±0.023^***^ | 0.001±0.0001^***^ |
| PL | DW | -0.03±0.001^***^ | 0.23±0.016^***^ | 0.21±0.022^***^ | 196.83±23.756^***^ |
|  | AW | -0.02±0.0008^***^ | -0.02±0.002^***^ | 0.23±0.023^***^ | -0.002±0.0001^***^ |
| Time*PL | DW | -0.005±0.002^*^ | -0.05±0.02^ns^ | -0.07±0.027^*^ | -145.69±29.096^**^ |
|  | AW | -0.006±0.001^**^ | 0.0008±0.002^ns^ | 0.05±0.028^ns^ | -0.0005±0.0002^ns^ |
| Time^2^ | DW | 0.008±0.002^**^ | -0.2±0.025^***^ | 0.02±0.034^ns^ | - |
|  | AW | 0.005±0.001^*^ | 0.003±0.003^ns^ | 0.91±0.035^***^ | -0.0002±0.0003^ns^ |
| PL^2^ | DW | 0.01±0.002^**^ | -0.08±0.025^*^ | -0.02±0.034^ns^ | - |
|  | AW | 0.01±0.001^***^ | 0.009±0.003^*^ | -0.03±0.035^ns^ | 0.0008±0.0003^*^ |
| R^2^/ Adjusted R^2^/  Predictive R^2^ | DW | 0.99/0.98/0.91 | 0.99/0.97/0.90 | 0.95/0.90/0.73 | 0.95/0.93/0.78 |
|  | AW | 0.99/0.99/0.97 | 0.98/0.95/0.83 | 0.99/0.99/0.98 | 0.98/0.95/0.77 |
| nRMSE (%) | DW | 5.60 | 4.30 | 6.80 | 6.10 |
|  | AW | 4.40 | 5.80 | 2.1 | 5.90 |
| Lack of fit  p value | DW | 0.18 | 0.14 | 0.45 | 0.06 |
|  | AW | 0.47 | 0.39 | 0.20 | 0.13 |
| Transformation | DW | 1/y | Log10 | Log10 | - |
|  | AW | 1/y | 1/y | Log10 | 1/y |

*Denotes significance at 0.05, ** denotes significance at 0.01, *** denotes significance at 0.001. Time = periods of incubation, PL = phosphorus level, DW = distilled water, AW = acidified water, BA = bone ash, BC = bone char, PR = phosphate rock, SSP = single super phosphate

**Table (S-1).** Regression coefficients ± standard error of the fitted model of available phosphorus (mg/kg) for the four sources of phosphorus using distilled water and acidified water

|  |  | BA | BC | PR | SSP |
| --- | --- | --- | --- | --- | --- |
| Intercept | DW | -0.6±0.064^***^ | 0.33±0.006^***^ | 4.19±0.231^***^ | 1.89±0.093^***^ |
|  | AW | 0.24±0.029^***^ | 1.02±0.129^***^ | -0.43±0.025^***^ | 1.05±0.366^*^ |
| Time | DW | -0.31±0.051^**^ | 0.05±0.004^***^ | -2.92±0.183^***^ | 0.26±0.074^*^ |
|  | AW | -0.2±0.023^***^ | -0.15±0.102^ns^ | 0.22±0.02^***^ | -2.23±0.291^***^ |
| PL | DW | 0.23±0.051^**^ | 0.13±0.004^***^ | -1.83±0.183^***^ | -0.81±0.074^***^ |
|  | AW | 0.3±0.023^***^ | -0.5±0.102^**^ | 0.19±0.02^***^ | 1.36±0.291^**^ |
| Time*PL | DW | -0.13±0.062^ns^ | 0.04±0.005^***^ | 0.57±0.225^ns^ | -0.08±0.09^ns^ |
|  | AW | -0.07±0.028^*^ | -0.32±0.125^ns^ | -0.0008±0.024^ns^ | -1.83±0.356^**^ |
| Time^2^ | DW | 0.27±0.078^*^ | -0.1±0.007^***^ | 2.16±0.282^***^ | -0.48±0.114^**^ |
|  | AW | -0.36±0.035^***^ | 0.8±0.157^**^ | -0.04±0.031^ns^ | 1.94±0.448^**^ |
| PL^2^ | DW | -0.02±0.078^ns^ | -0.02±0.007^*^ | 0.41±0.282^ns^ | 0.29±0.114^*^ |
|  | AW | -0.09±0.035^ns^ | 0.17±0.157^ns^ | 0.003±0.031^ns^ | 0.04±0.448^ns^ |
| R^2^/ Adjusted R^2^/  Predictive R^2^ | DW | 0.94/0.88/0.41 | 0.99/0.99/0.99 | 0.99/0.98/0.91 | 0.97/0.94/0.73 |
|  | AW | 0.99/0.97/0.87 | 0.93/0.86/0.40 | 0.98/0.96/0.82 | 0.96/0.92/0.61 |
| nRMSE (%) | DW | 5.30 | 2.00 | 5.10 | 5.50 |
|  | AW | 4.30 | 15.5 | 4.40 | 5.70 |
| Lack of fit  p value | DW | 0.06 | 0.91 | 0.34 | 0.29 |
|  | AW | 0.07 | 0.07 | 0.41 | 0.04 |
| Transformation | DW | Log10 | - | 1/y | 1/y |
|  | AW | Log10 | 1/sqrt(y) | Log10 | - |

*Denotes significance at 0.05, ** denotes significance at 0.01, *** denotes significance at 0.001. Time = periods of incubation, PL = phosphorus level. DW = distilled water, AW = acidified water, BA = bone ash, BC = bone char, PR = phosphate rock, SSP = single super phosphate.

**Table (S-2).** Regression coefficients ± standard error of the fitted model of soluble phosphorus (mg/kg) for the four sources of phosphorus using distilled water and acidified water

|  |  | BA | BC | PR | SSP |
| --- | --- | --- | --- | --- | --- |
| Intercept | DW | 0.34±0.008^***^ | 2.72±0.096^***^ | 0.54±0.018^***^ | 0.06±0.005^***^ |
|  | AW | 0.41±0.012^***^ | 0.48±0.015^***^ | 0.11±0.005^***^ | 0.21±0.003^***^ |
| Time | DW | -0.14±0.007^***^ | 0.57±0.076^***^ | 0.03±0.014^ns^ | -0.004±0.004^ns^ |
|  | AW | -0.12±0.009^***^ | -0.2±0.012^***^ | -0.07±0.004^***^ | -0.02±0.002^***^ |
| PL | DW | -0.12±0.007^***^ | 0.47±0.076^**^ | -0.14±0.014^***^ | -0.04±0.004^***^ |
|  | AW | -0.06±0.009^**^ | -0.04±0.012^*^ | -0.05±0.004^***^ | -0.04±0.002^***^ |
| Time*PL | DW | 0.09±0.008^***^ | 0.05±0.093^ns^ | -0.03±0.018^ns^ | -0.01±0.004^ns^ |
|  | AW | 0.03±0.012^*^ | 0.01±0.015^ns^ | 0.03±0.005^***^ | 0.0007±0.003^ns^ |
| Time^2^ | DW | 0.04±0.01^*^ | -0.76±0.117^**^ | 0.06±0.022^*^ | 0.001±0.006^ns^ |
|  | AW | 0.06±0.014^**^ | 0.13±0.019^***^ | 0.04±0.006^**^ | 0.01±0.004^*^ |
| PL^2^ | DW | 0.1±0.01^***^ | -0.08±0.117^ns^ | 0.04±0.022^ns^ | 0.02±0.006^*^ |
|  | AW | 0.01±0.014^ns^ | -0.02±0.019^ns^ | 0.04±0.006^**^ | 0.007±0.004^ns^ |
| R^2^/ Adjusted R^2^/  Predictive R^2^ | DW | 0.99/0.99/0.99 | 0.97/0.93/0.80 | 0.96/0.92/0.81 | 0.97/0.94/0.69 |
|  | AW | 0.98/0.96/0.80 | 0.99/0.97/0.87 | 0.99/0.99/0.94 | 0.99/0.98/0.89 |
| nRMSE (%) | DW | 3.10 | 6.30 | 10.50 | 9.40 |
|  | AW | 5.50 | 6.50 | 6.80 | 3.60 |
| Lack of fit  p value | DW | 0.94 | 0.32 | 0.80 | 0.10 |
|  | AW | 0.14 | 0.07 | 0.053 | 0.07 |
| Transformation | DW | 1/y | - | 1/sqrt(y) | 1/y |
|  | AW | 1/sqrt(y) | 1/sqrt(y) | 1/y | 1/sqrt(y) |

*Denotes significance at 0.05, ** denotes significance at 0.01, *** denotes significance at 0.001. Time = periods of incubation, PL = phosphorus level. DW = distilled water, AW = acidified water, BA = bone ash, BC = bone char, PR = phosphate rock, SSP = single super phosphate.

**Table (S-3).** Regression coefficients ± standard error of the fitted model of soluble calcium (mg/kg) for the four sources of phosphorus using distilled water and acidified water

|  |  | BA | BC | PR | SSP |
| --- | --- | --- | --- | --- | --- |
| Intercept | DW | 1.91±0.093^***^ | 0.25±0.019^***^ | 1.37±0.069^***^ | 0.72±0.027^***^ |
|  | AW | 0.34±0.017^***^ | 4.01±0.114^***^ | 0.51±0.021^***^ | 6.93±0.866^***^ |
| Time | DW | -0.75±0.074^***^ | 0.19±0.015^***^ | 0.23±0.094^*^ | 0.09±0.036^*^ |
|  | AW | -0.09±0.013^**^ | 0.21±0.155^ns^ | 0.17±0.017^***^ | 3.5±1.172^*^ |
| PL | DW | 0.37±0.074^**^ | 0.01±0.015^ns^ | 0.53±0.094^***^ | 0.16±0.036^**^ |
|  | AW | -0.13±0.013^***^ | -0.66±0.155^**^ | 0.11±0.017^**^ | 0.6±1.172^ns^ |
| Time*PL | DW | -0.45±0.091^**^ | 0.02±0.018^ns^ | -0.1±0.115^ns^ | -0.07±0.044^ns^ |
|  | AW | 0.06±0.016^*^ | - | -0.07±0.02^*^ | - |
| Time^2^ | DW | -0.44±0.114^*^ | -0.16±0.023^***^ | - | - |
|  | AW | -0.04±0.021^ns^ | - | 0.07±0.026^*^ | - |
| PL^2^ | DW | -0.12±0.114^ns^ | -0.01±0.023^ns^ | - | - |
|  | AW | 0.06±0.021^*^ | 2.85±0.379^***^ | -0.08±0.026^*^ | - |
| R^2^/ Adjusted R^2^/  Predictive R^2^ | DW | 0.97/0.94/0.82 | 0.98/0.96/0.85 | 0.85/0.78/0.63 | 0.80/0.71/0.23 |
|  | AW | 0.97/0.94/0.76 | 0.92/0.88/0.78 | 0.97/0.94/0.81 | 0.53/0.42/0.21 |
| nRMSE (%) | DW | 5.10 | 6.60 | 11.4 | 15.20 |
|  | AW | 8.90 | 8.80 | 6.50 | 22.10 |
| Lack of fit  p value | DW | 0.63 | 0.11 | 0.17 | 0.08 |
|  | AW | 0.38 | 0.24 | 0.23 | 0.06 |
| Transformation | DW | - | Log10 | - | Log10 |
|  | AW | 1/y | - | Log10 | - |

*Denotes significance at 0.05, ** denotes significance at 0.01, *** denotes significance at 0.001. Time = periods of incubation, PL = phosphorus level. DW = distilled water, AW = acidified water, BA = bone ash, BC = bone char, PR = phosphate rock, SSP = single super phosphate.

**Table (S-4).** Regression coefficients ± standard error of the fitted model of soluble magnesium (mg/kg) for the four sources of phosphorus using distilled water and acidified water

|  |  | BA | BC | PR | SSP |
| --- | --- | --- | --- | --- | --- |
| Intercept | DW | 8.4±0.012^***^ | 0.19±0.00012^***^ | 8.17±0.008^***^ | 7.77±0.018^***^ |
|  | AW | 8.18±0.024^***^ | 8.11±0.039^***^ | 8.02±0.013^***^ | 7.66±0.029^***^ |
| Time | DW | -0.01±0.01^ns^ | -0.0001±0.00009^ns^ | -0.01±0.011^ns^ | 0.09±0.014^**^ |
|  | AW | -0.09±0.032^*^ | -0.1±0.031^*^ | -0.15±0.018^***^ | 0.02±0.023^ns^ |
| PL | DW | 0.06±0.01^**^ | -0.0004±0.00009^**^ | -0.04±0.011^*^ | -0.13±0.014^***^ |
|  | AW | 0.1±0.032^*^ | 0.02±0.031^ns^ | -0.01±0.018^ns^ | -0.18±0.023^***^ |
| Time*PL | DW | -0.007±0.012^ns^ | -0.0003±0.000^ns^ | 0.05±0.014^**^ | 0.02±0.017^ns^ |
|  | AW | - | -0.05±0.038^ns^ | - | 0.14±0.029^**^ |
| Time^2^ | DW | 0.16±0.015^***^ | -0.0011±0.0001^***^ | - | 0.03±0.022^ns^ |
|  | AW | - | 0.25±0.048^**^ | - | -0.16±0.036^**^ |
| PL^2^ | DW | -0.02±0.015^ns^ | -0.00002±0.0001^ns^ | - | -0.06±0.022^*^ |
|  | AW | - | -0.12±0.048^ns^ | - | 0.06±0.036^ns^ |
| R^2^/ Adjusted R^2^/  Predictive R^2^ | DW | 0.97/0.94/0.86 | 0.94/0.89/0.46 | 0.79/0.70/0.48 | 0.97/0.93/0.70 |
|  | AW | 0.68/0.61/0.40 | 0.89/0.78/0.35 | 0.89/0.87/0.80 | 0.95/0.91/0.73 |
| nRMSE (%) | DW | 5.50 | 6.60 | 12.40 | 4.70 |
|  | AW | 14.90 | 9.90 | 10.00 | 5.40 |
| Lack of fit  p value | DW | 0.83 | 0.07 | 0.25 | 0.15 |
|  | AW | 0.01 | 0.25 | 0.60 | 0.50 |
| Transformation | DW | - | 1/y | - | - |
|  | AW | - | - | - | - |

*Denotes significance at 0.05, ** denotes significance at 0.01, *** denotes significance at 0.001. Time = periods of incubation, PL = phosphorus level. DW = distilled water, AW = acidified water, BA = bone ash, BC = bone char, PR = phosphate rock, SSP = single super phosphate.

**Table (S-5).** Regression coefficients ± standard error of the fitted model of pH for the four sources of phosphorus using distilled water and acidified water
